# Identification of collagen genes related to immune infiltration and epithelial-mesenchymal transition in glioma

**DOI:** 10.1186/s12935-021-01982-0

**Published:** 2021-05-25

**Authors:** Wen Yin, Hecheng Zhu, Jun Tan, Zhaoqi Xin, Quanwei Zhou, Yudong Cao, Zhaoping Wu, Lei Wang, Ming Zhao, Xingjun Jiang, Caiping Ren, Guihua Tang

**Affiliations:** 1 Department of Neurosurgery, Xiangya Hospital of Central South University, Changsha, Hunan Province 410008 China; 2 Changsha Kexin Cancer Hospital, Changsha, Hunan 410205 China; 3 Cancer Research Institute, Collaborative Innovation Center for Cancer Medicine, The Key Laboratory for Carcinogenesis of Chinese Ministry of Health and the Key Laboratory of Carcinogenesis and Cancer Invasion of the Chinese Ministry of Education, School of Basic Medical Science, Central South University, Changsha, Hunan People’s Republic of China; 4 Department of Clinical Laboratory, Hunan Provincial People’s Hospital (The first affiliated hospital of Hunan Normal University, The college of clinical medicine of Human Normal University), Changsha, Hunan Province 410005 China

**Keywords:** Glioma, Collagen gene, Weighted gene co-expression network analysis (WGCNA), Prognostic markers, Epithelial-mesenchymal transition (EMT), Immune microenvironment

## Abstract

**Background:**

Gliomas account for the majority of fatal primary brain tumors, and there is much room for research in the underlying pathogenesis, the multistep progression of glioma, and how to improve survival. In our study, we aimed to identify potential biomarkers or therapeutic targets of glioma and study the mechanism underlying the tumor progression.

**Methods:**

We downloaded the microarray datasets (GSE43378 and GSE7696) from the Gene Expression Omnibus (GEO) database. Then, we used weighted gene co-expression network analysis (WGCNA) to screen potential biomarkers or therapeutic targets related to the tumor progression. ESTIMATE (Estimation of STromal and Immune cells in MAlignant Tumors using Expression data) algorithm and TIMER (Tumor Immune Estimation Resource) database were used to analyze the correlation between the selected genes and the tumor microenvironment. Real-time reverse transcription polymerase chain reaction was used to measure the selected gene. Transwell and wound healing assays were used to measure the cell migration and invasion capacity. Western blotting was used to test the expression of epithelial-mesenchymal transition (EMT) related markers.

**Results:**

We identified specific module genes that were positively correlated with the WHO grade but negatively correlated with OS of glioma. Importantly, we identified that 6 collagen genes (COL1A1, COL1A2, COL3A1, COL4A1, COL4A2, and COL5A2) could regulate the immunosuppressive microenvironment of glioma. Moreover, we found that these collagen genes were significantly involved in the EMT process of glioma. Finally, taking COL3A1 as a further research object, the results showed that knockdown of COL3A1 significantly inhibited the migration, invasion, and EMT process of SHG44 and A172 cells.

**Conclusions:**

In summary, our study demonstrated that collagen genes play an important role in regulating the immunosuppressive microenvironment and EMT process of glioma and could serve as potential therapeutic targets for glioma management.

**Supplementary Information:**

The online version contains supplementary material available at 10.1186/s12935-021-01982-0.

## Introduction

Gliomas are the most fatal primary brain tumors [1]. There were about 30 % central nervous system (CNS) tumors diagnosed as gliomas in the United States, and even worse, this data was as high as 81 % in malignant CNS tumors [2]. The World Health Organization (WHO) classified gliomas into grades I–IV based on histological characteristics, which contained increased degrees of anaplasia, undifferentiation, and infiltration [3]. Diffuse low-grade (WHO grade II) and III intermediate-grade gliomas are called lower-grade gliomas (LGGs) [4]. Because of their rapid growth and highly remarkable aggressiveness, a subset of these LGGs will develop into glioblastoma (WHO grade IV) within several months [5]. Glioblastoma (GBM) is the most common and malignant glioma, which is characterized by poor clinical prognosis and the survival time rarely exceeds 14 months [6].

Unfortunately, despite some remarkable achievements, the median survival time of glioma patients has not been significantly prolonged in the past decades [5]. There is still much room for research in the underlying pathogenesis, the multistep progression of glioma, and how to improve survival. Malignant gliomas are diffusely infiltrated into the surrounding normal brain tissue. Brain extracellular matrix (ECM) plays a crucial role in modulating and driving glioma invasion [7]. The ECM comprises numerous proteins, including collagen, proteoglycans, laminin, and fibronectin [8]. Collagen is the most important component of the ECM acting as a scaffold to provide sites for tumor cell adhesion [8]. Few studies have focused on the relationship between collagen genes and glioma; more studies are needed to elucidate the function of collagen genes in glioma progression.

Weighted gene co-expression network (WGCNA) is a powerful systems biology method to identify potential biomarkers or therapeutic targets and study the mechanism underlying the tumor progression involved [9, 10]. In this study, we performed WGCNA to identify the clusters of highly interconnected genes that correlated with WHO grades and overall survival, and we found such module and intramodular hub genes by facilitating network-based gene screening methods. Then, six collagen genes (COL1A1, COL1A2, COL3A1, COL4A1, COL4A2, and COL5A2) were selected for further validation in Oncomine, TCGA (The Cancer Genome Atlas), and CGGA (Chinese Glioma Genome Atlas) database. Further analysis revealed that the expression of the collagen genes is positively related to stromal and immune scores, immunosuppressive cell recruitment and immunosuppressive factors, and the infiltration of various immune cells. Moreover, our study also demonstrated that the collagen genes were significantly involved in the epithelial-mesenchymal transition (EMT) process, which was closely related to glioma malignancies. Finally, taking COL3A1 as a further research object, the result showed that knockdown COL3A1 could significantly inhibit the migration, invasion, and EMT process of glioma cells *in vitro*. These results may provide a novel understanding of collagen genes in glioma progression. At the same time, we also found that collagen genes may become promising therapeutic targets for fighting against gliomas.

## Materials and methods

### Data collection

The gliomas mRNA expression microarrays were downloaded from the repository browser of the Gene Expression Omnibus (GEO) database (https://www.ncbi.nlm.nih.gov/geo/summary/). We selected such datasets of which the presented sample information contained WHO grades and survival time. Besides, datasets with a sample size of less than 30 were removed to minimize bias resulted from small samples. Ultimately, datasets with the accession number of GSE43378 [11] and GSE7696 [12, 13] performed on the Affymetrix Human Genome U133 Plus 2.0 Array platform (GPL570) were downloaded. The raw data (CEL files and annotation files) of this platform and related sample information were also obtained. There were a total of 120 glioma samples and 4 non-tumor specimens after eliminating recurrent samples. Moreover, TCGA GBMLGG dataset (dataset ID: TCGA.GBMLGG.sampleMap/HiSeqV2) downloaded from the UCSC Xena database (https://xena.ucsc.edu/public) and CCGA glioma dataset (https://www.cgga.org.cn/; dataset ID: mRNAseq_325) were used as validation datasets in this study.

### Differentially expressed genes (DEGs) screening

We applied the R package “affy” [14] for preprocessing expression microarrays. Briefly, original files were normalized using the Robust-Multi Array (RMA) method followed by logarithm transformed based on 2. Subsequently, another R package “sva” [15] was used to adjust batch effects between different datasets and the “limma” [16] package to screen differentially expressed genes (DEGs) between gliomas and non-tumor controls. The cut-off criteria of DEGs were defined as log_2_ fold-change (log_2_FC) more than 1 or less than − 1 with false discovery rate (FDR) adjusted *p*-value less than 0.05.

### Gene co-expression network construction

A weighted gene co-expression network of DEGs was constructed by using an R package “WGCNA” [9]. Outlier samples were eliminated by clustering analysis before WGCNA analysis to ensure network reliability. A suitable soft threshold power of 8 with a model fitting index R^2^ > 0.9 was selected to maximize scale-free topology and the adjacencies matrix of topology similarity were calculated applying a power function, following, transformed to a topological overlap matrix (TOM). Then, the corresponding dissimilarity (1-TOM) was also calculated as the distance to hierarchically cluster genes, with which the modules were identified by the dynamic tree cut method and resulted to dendrogram. The module eigengene (ME) and module membership (MM) were calculated by function moduleEigengenes and signedKME respectively. ME was considered as the first principal component of a clustered module representing the gene expression profiles, and MM correlated ME with gene expression values, so it quantified the membership of a gene with respect to a given module.

### Identifying significant modules associated with clinical traits

MEs were applied to evaluate the correlations between identified modules and clinical traits including WHO grade and survival time. It was considered statistically significantly correlated when the *p*-value of Pearson’s correlation tests was no more than 0.05. The module was identified as the candidate that of the most outstanding correlation coefficients and significant correlations with clinical traits. For further analysis, the gene significance (GS) was defined as the correlation between individual genes and clinical traits. Generally, GS and MM were highly associated, meaning that the genes were highly important elements and significantly correlated with clinical traits.

### GO terms and KEGG pathways

Gene ontology (GO) and Kyoto Encyclopedia of Genes and Genomes (KEGG) enrichment analysis of candidate module genes were performed to understand the biological functions [17]. GO terms which included cellular components (CC), biological processes (BP), and molecular function (MF) were achieved in the Database for Annotation, Visualization, and Integrated Discovery (DAVID), and KEGG pathway enrichment analysis was accomplished in R software using KEGG package. The enrichment analysis was considered as significant only when the FDR < 0.05.

### Screening and validation of the collagen genes

The co-expression network of the candidate module was imported to Cytoscape [18] software (v3.6.1), which is powerful for integrating and visualization biomolecular interaction networks. Hub genes that highly connected intramodule were determined by cytoHubba. Genes that positively correlated with glioma grade and with a significant *p* < 0.05 in grade plots and survival analysis were defined as the “actual” hub genes. Six collagen genes were identified from the hub genes for further analysis.

Subsequently, to determine the expression level of collagen genes in different cancer types and validate the relationship between candidate collagen genes and glioma grades, online expression analysis was performed on the database Oncomine [19] (https://www.oncomine.org/). Simultaneously, the Chinese Glioma Genome Atlas (CGGA) dataset was used to not only validate the relationship between candidate gene expression and WHO grades, but also molecular subgroup based on the mutation status of IDH and codeletion status of 1p/19q. Moreover, GBMLGG RNA-seq datasets in The Cancer Genome Atlas (TCGA) database was download from UCSC Xena (https://xena.ucsc.edu/) to validate the relationship between candidate gene expression and WHO grades, and overall survival (OS) analyses were also completed in the Gene Expression Profiling Interactive Analysis (GEPIA) [20] database to display the prognostic values of these genes. Finally, the protein-level validation of the collagen genes was carried out in the Human Protein Atlas database (https://www.proteinatlas.org/).

### Analysis of immune infiltration characteristics

ESTIMATE (Estimation of STromal and Immune cells in MAlignant Tumors using Expression data) is a method that uses gene expression profile to infer the fraction of stromal and immune cells in tumor samples [21]. Three scores are generated by the ESTIMATE algorithm: stromal score (reflecting the infiltration of stromal cells in tumor samples), immune score (reflecting the infiltration of immune cells in tumor samples) and estimate score (inferring tumor purity in tumor samples). According to a previous study, immunosuppressive cell recruitment factors and immunosuppressive factors were selected for further analysis [22]. Then the correlation between collagen expressions and immunosuppressive cell recruitment factor and immunosuppressive factors were analyzed by R language.

### Associations between the collagen genes and tumor immune infiltrating cells

Tumor Immune Estimation Resource (TIMER) database (http://cistrome.dfci.harvard.edu/TIMER/) is an online tool for assessing the association between the specific gene(s) and tumor immune infiltrating cells [23]. 8 tumor immune infiltrating cells (B-cells, CD4 + T-cells, CD8 + T-cells, dendritic cells, macrophages, and neutrophils) were included in the TIMER database. By using the TIMER database, we explored the correlations between the collagen genes and tumor immune infiltrating cells in both LGGs and GBM.

### The correlation between EMT markers and the collagen genes

10 EMT-related genes (TJP1, CDH1, CDH2, FN1, VIM, CTNNB1, TWIST1, SNAI2, SNAI1, and ACTA2) were selected from published literature [22]. Based on TCGA database, Pearson correlation coefficients between EMT markers and collagen genes were calculated. Pearson correlation coefficient reflects the degree of linear correlation between the two genes.

### Protein-protein interaction (PPI) network construction

The 6 collagen genes and 10 EMT-related genes were imported into the STRING database (https://string-db.org/), which is a web tool used to explore protein-protein interactions.

### Cell culture and real-time quantitative PCR (qRT-PCR)

SHG44 and A172 glioma cells were provided by Xiangya Medical School of Central South University, Changsha, China. The SHG44 and A172 cells were cultured in DMEM high glucose medium (Gibco/Thermo Fisher Scientifc, Inc.) with 10 % fetal bovine serum. All cells were maintained at 37 °C with a humidified atmosphere of 5 % CO2. The siRNAs against the COL3A1 gene were synthesized by RiboBio Corporation (Guangzhou, China). The siCOL3A1 target sequence utilized in this experiment was the following: CUAUGCGGAUAGAGAUGUCTT.

Total RNA was extracted from treated SHG44 and A172 cells by the Trizol lysis method. cDNA synthesis was performed by using the Thermo Scientific RevertAid First Strand cDNA Synthesis Kit (Thermo Scientific, Waltham, MA). RNA levels of COL3A1 were detected by using qRT-PCR according to the manufacturer’s protocol. Expression of COL3A1 and GAPDH were analyzed by the 2^−ΔΔCt^ method. The primers were produced by Sangon (Shanghai, China) and the sequences were designed as follows: for COL3A1, the forward primer was 5’-GGAGCTGGCTACTTCTCGC-3’ and the reverse primer was 5’-GGGAACATCCTCCTTCAACAG-3’. For GAPDH, the forward primer was 5’-CATTGACCTCAACTACATGGTT-3’ and the reverse primer was 5’-CCATTGATGACAAGCTTCCC-3’.

### **Wound healing and transwell assays**

Wound healing and transwell assay were determined as previously described [24].

### **Western blotting assay**

Western blotting assay was conducted as our previous study described [24]. Antibodies against GPDH (10494-1-AP, Proteintech), Vimentin (10366-1-AP, Proteintech), and N-cadherin (22018-1-AP, Proteintech) were used in the Western blotting.

### **Immunofluorescence staining**

The experiment was approved by the Human Ethics Committee of Xiangya Hospital (Changsha, China), and informed consent was obtained from all patients. Glioma specimens were fixed with 4 % paraformaldehyde for 72 h, and then sectioned and subjected to sodium citrate antigen retrieval. Slides were incubated in PBS (containing 5 % BSA and 0.1 % Triton X-100) for 1 h at RT. After being incubated with primary antibodies COL1A1 (67288-1-Ig, Proteintech) and IL-6 (67288-1-Ig, Proteintech) at 4 °C overnight, the slides were washed 3 times and then incubated in secondary antibody (Abcam, United States) 1 h at RT. DAPI (Sigma, United States) was used to stain the Cell nuclei. Olympus BX51was used to capture the images.

### **Statistical analysis**

Statistical analyses were performed using the R software (version 4.0.0) and GraphPad Prism (version 7.0.0). The log-rank test was used in the Kaplan-Meier survival analysis. The coefficients of correlation were calculated by Pearson correlation analysis. Differences in quantitative data between the two groups were compared by using Student’s t-test. *p < 0.05, **p < 0.01, ***p < 0.001 or ****p < 0.0001 was considered to be significant.

## Results

### WGCNA identified clinically significant modules

**Fig. 1:**
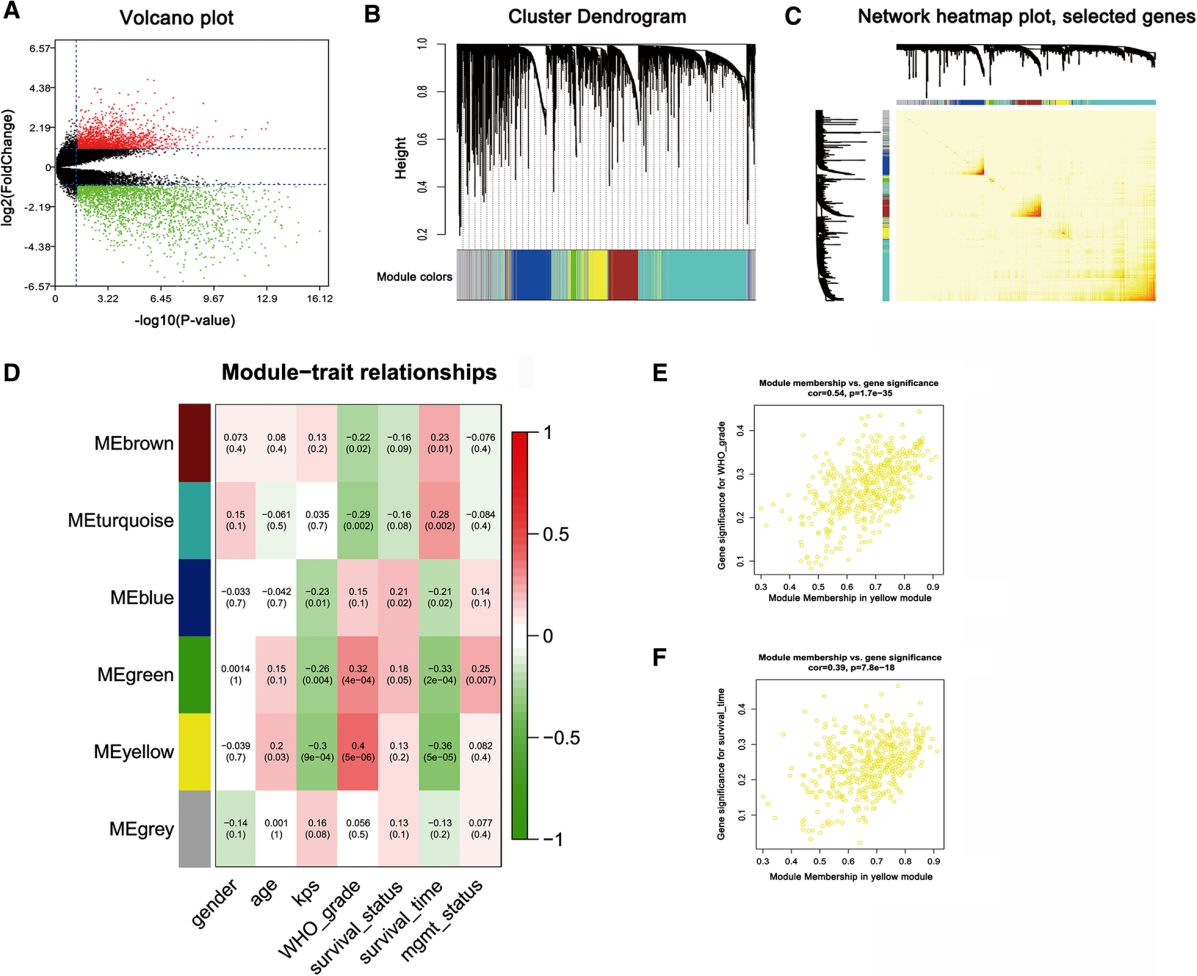
Identification of co-expression gene modules of glioma by WGCNA. **A** Volcano plots showing the DEGs between glioma samples and non-tumor samples. Red dots indicate up-regulated genes, while green dots down-regulated genes. **B** Cluster dendrogram of different gene modules. Genes were clustered into a module of high interconnection and marked by different colors in the horizontal bar (grey represented unassigned genes). **C** Network heatmap plot. Each column and row represent genes, low topological overlap showed light colors and higher topological overlap showed darker colors. **D** Relationships between modules and clinical traits. Each column corresponds to a clinical trait, and the row corresponds to a module eigengene. Each unit includes the corresponding correlation coefficient and *p*-value in the first and second lines respectively. **E**, **F** indicate that the yellow module significantly correlated with glioma WHO-grade and survival time. Each dot represents a unique gene within the module, which was plotted by MM on the x-axis and GS on the y-axis. Besides, the correlation value and p-value were displayed

In total, 120 primary glioma samples and 4 non-tumor samples were obtained from the GSE43378 and GSE7696. After data preprocessing and normalization, 4219 DEGs were selected for analysis, including 2340 down-regulated and 1879 up-regulated in glioma samples. Expression profiling was presented in a volcano plot (Fig. 1A).

Identified DEGs were submitted to WGCNA, which were assigned to gene co-expression modules after removing outlier samples by cluster dendrogram trees (Fig. 1B). The co-expression network clustered to 7 modules of at least 30 genes, corresponding to 7 different colors. Module-containing genes were assigned by their intramodule connectivity and the non-clustering genes were divided into the gray module. TOM reflecting adjacencies or topological overlaps was visualized by heatmap (Fig. 1C), the topological overlap depicted the similar commonality between notes interconnected. It can be known from the topological overlap heatmap that more obvious topological overlap appeared in genes within a module than across modules.

As displayed in the relationship between modules and clinical traits (Fig. 1D), the yellow module was the most highlighted. It was not only positively correlated with WHO grade (correlation coefficient = 0.4, *p* = 5e-06) but also negative correlated with survival (correlation coefficient = − 0.36, *p* = 5e-05). It was suggested that the yellow module genes played a role in promoting the development of glioma and leading to a poor prognosis. Subsequently, for further investigating the correlations between the yellow module and clinical traits, the relationships between GS and MM in the module were plotted to verify the significance of yellow module related to WHO grade (Fig. 1E) and survival (Fig. 1F).

### Screening collagen genes

401 genes in the yellow module were submitted to DAVID website to enrich their potential functions. The results showed enriched BP (Additional file 1: Fig. S1A) was mainly focused on extracellular matrix (ECM) organization, cell adhesion, collagen fibril organization, angiogenesis, leukocyte migration, and single organismal cell-cell adhesion. MF (Additional file 1: Fig. S1B) mainly concentrated on ECM structural constituent, the binding of biomolecules (including protein, platelet-derived growth factor, integrin, and collagen), along with cadherin binding involved in cell-cell adhesion. With regards to CC (Additional file 1: Fig. S1C), a variety of enrichments were mainly involved in extracellular exosome, extracellular matrix, focal adhesion, and so on. The results of KEGG enrichment analysis (Additional file 1: Fig. S1D) indicated that the tumor-associated pathways in the yellow module were closely associated with ECM-receptor interaction, focal adhesion, protein processing in the endoplasmic reticulum, and PI3K-Akt signaling pathway. Based on these results, we hypothesized that genes correlated with the ECM-receptor interaction signal pathway may play an important role in glioma progression.

We then imported the weighted co-expression networks of the yellow module to Cytoscape followed by screening the candidate hub genes, which are keynotes in the interactive network. A total of four calculation methods, including Degree, Edge percolated component (EPC), Closeness, and Radiality in cytoHubba application were employed in selecting hub genes, which were the intersection of the top 20 genes determined by these algorithms (Table 1). Eventually, 6 collagen genes (COL1A1, COL1A2, COL3A1, COL4A1, COL4A2, and COL5A2) in a family were identified as the candidate key genes for further verification. And the weighted co-expression network of these genes interacting within the module was visualized (Additional file 1: Fig. S1E).

**Table 1:** Hub genes ranked in cytoHubba

Category	Rank methods in cytoHubba			
	Degree	EPC	Closeness	Radiality
Genes top 20	FN1	FN1	FN1	FN1
	CD44	**COL4A1**	**COL4A1**	**COL4A1**
	**COL4A1**	ANGPT2	ANXA2	ANXA2
	ANXA2	**COL5A2**	CD44	ANGPT2
	**COL5A2**	ANXA2	ANGPT2	LAMB1
	ANGPT2	**COL3A1**	LAMB1	**COL1A1**
	**COL3A1**	**COL1A1**	SERPINH1	SERPINH1
	**COL1A1**	CALD1	**COL5A2**	CALD1
	CLIC1	CD44	**COL1A1**	CD44
	CALD1	SERPINH1	CALD1	NAMPT
	LAMB1	**COL1A2**	**COL1A2**	LAMC1
	SERPINH1	NAMPT	LAMC1	**COL5A2**
	**COL1A2**	LAMB1	NAMPT	**COL1A2**
	LAMC1	LAMC1	CLIC1	PDPN
	NAMPT	CLIC1	**COL3A1**	**COL4A2**
	ADAM12	**COL4A2**	**COL4A2**	GALNT2
	CAV1	ADAM12	PDPN	P4HB
	**COL4A2**	CAV1	P4HB	CXCR4
	CD93	NOX4	ADAM12	CLIC1
	PDPN	ENPEP	GALNT2	**COL3A1**

Top 20 genes are determined by six algorithms respectively in cytoHubba. The bold ones represent selected collagen genes;* EPC* Edge percolated component

### Higher expression of 6 collagen genes was negatively correlated with the prognosis of glioma patients

To test the collagen genes, differential expression analyses among different cancers and glioma grades were executed on a cancer microarray data-mining database: Oncomine [25]. The results showed that the collagen genes were overexpressed in most types of tumors, including central nervous system cancer, breast cancer, head and neck cancer, colorectal cancer, and so on (Additional file 2: Fig. S2A). Further, we utilized the Sun Brain dataset [26], a maximal sample size mRNA microarray that could be grouped by glioma grade in histology analysis. The grade plots of collagen genes (Additional file 2: Fig. S2B) were completed. The screened collagen genes were almost high-expression in glioma, and the expression level rises with glioma grade increases.

**Fig. 2:**
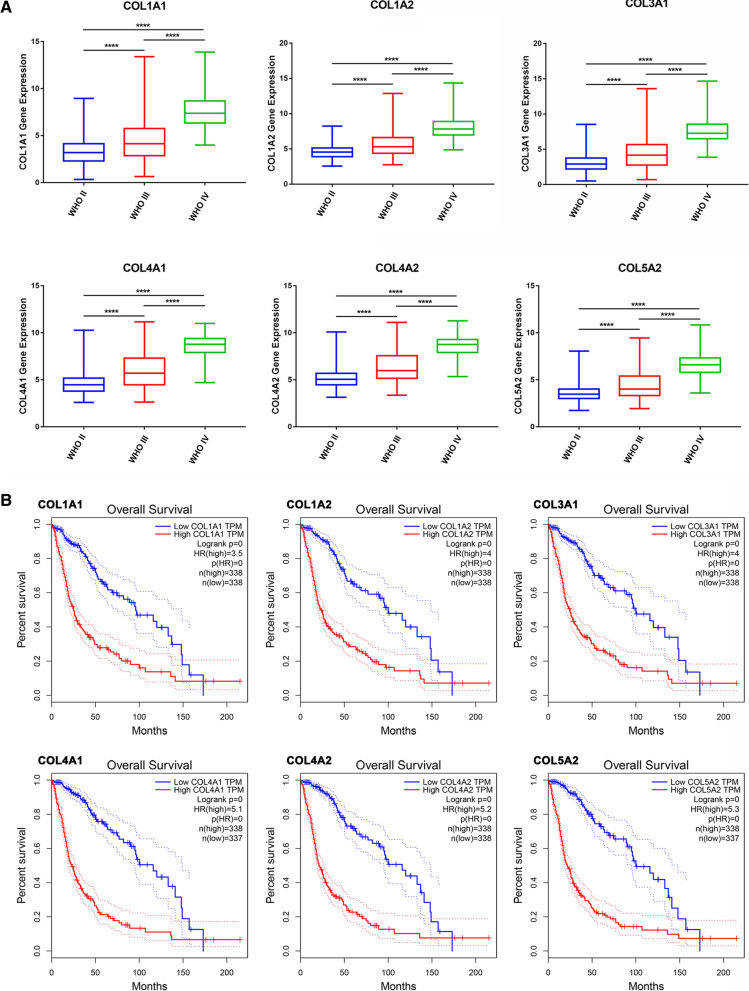
Increased COL1A1, COL1A2, COL3A1, COL4A1, COL4A2, and COL5A2 mRNA levels correlate with poor survival rates in glioma patients. **A** COL1A1, COL1A2, COL3A1, COL4A1, COL4A2, and COL5A2 mRNA levels were positively correlated with WHO grades based on TCGA database. **B** Kaplan-Meier analysis of the relationships between collagen gene expression and OS time in glioma (performed in GEPIA database). The red line represents gene high expression in samples, and blue indicates low expression

From the UCSC database, level 3 RNA-Seq datasets (TCGA-GBMLGG) and their clinical information were downloaded to investigate the relationship between gene expressions and WHO grades of patients. As showed in Fig. 2A, similar results were achieved. Besides, survival analyses were performed on the GEPIA database, which was based on The Cancer Genome Atlas (TCGA) data, to confirm the prognostic value of candidate genes. We found that high expression of collagen genes showed a significantly poor prognosis (Fig. 2B). On the CGGA website (mRNAseq_325), collagen genes (COL1A1, COL1A2, COL3A1, COL4A1, COL4A2, and COL5A2) in WHO grades II, III, and IV glioma samples were analyzed to explore the expression levels of these genes. Similarly, higher mRNA levels of these collagen genes were also found in higher grade glioma (Additional file 3: Fig. S3A–F).

**Fig. 3:**
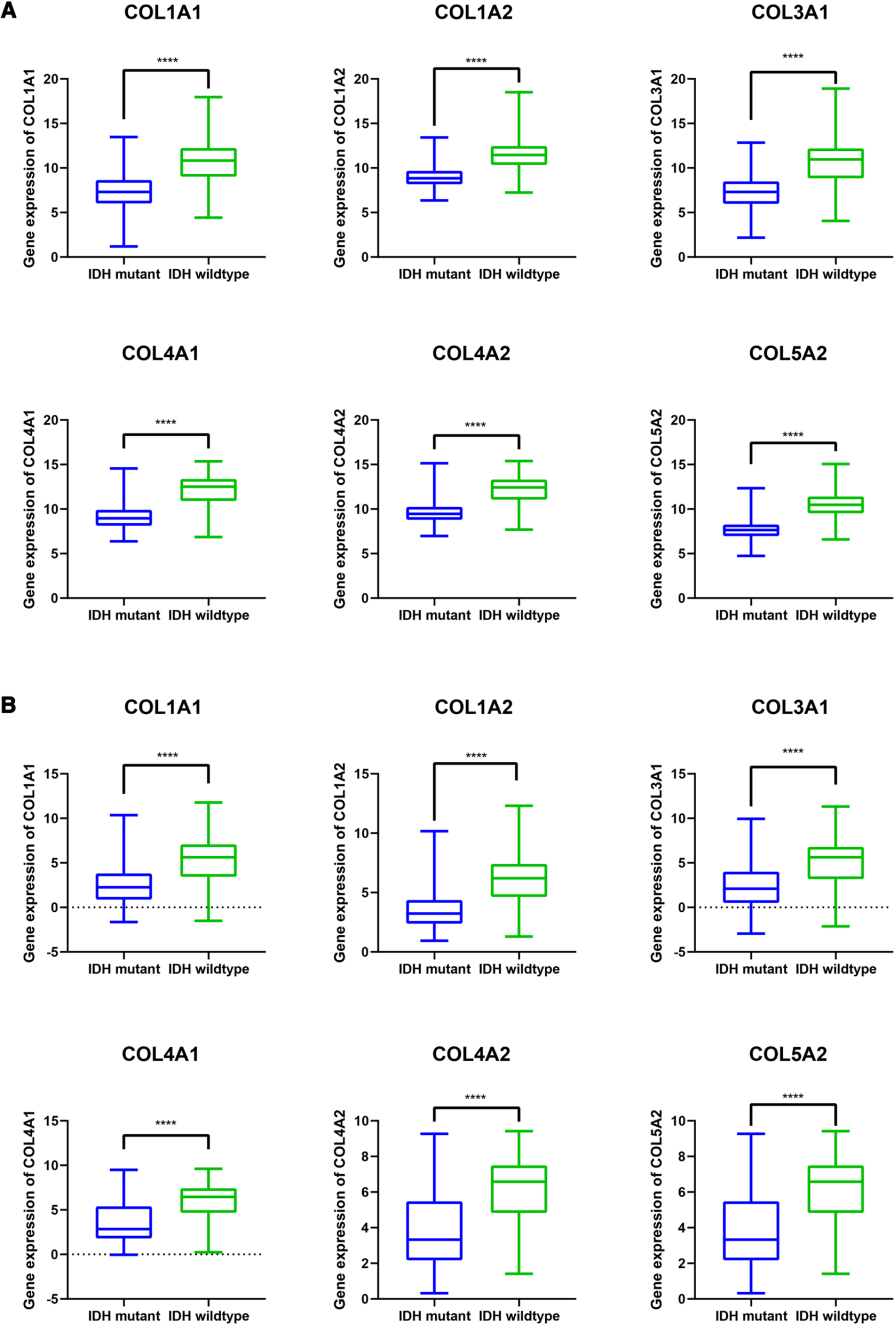
The mRNA expression levels of collagen genes in IDH mutant and wildtype glioma of TCGA and CGGA datasets. The expression of COL1A1, COL1A2, COL3A1, COL4A1, COL4A2, and COL5A2 in IDH mutant and IDH wild-type subgroups of TCGA (**A**) and CGGA (**B**) datasets. **** *p* < 0.0001

As we all know, IDH wild-type glioma patients present a shorter progression-free survival (PFS) and overall survival than mutant-type patients [27]. Subsequently, we found that glioma patients with IDH mutant-type have lower expression levels of these collagen genes than IDH wild-type patients in TCGA dataset (Fig. 3A). Moreover, there were similar findings in the CGGA dataset (Fig. 3B). These results indicated that the expressions of the 6 collagen genes were negatively correlated with the prognosis of glioma patients, and may become an independent prognostic marker.

Moreover, immunohistochemistry (IHC) datasets retrieved from the Human Protein Atlas database were utilized to reveal the protein level of the collagen gene. In total, four collagen genes (COL1A1, COL1A2, COL4A1, and COL4A2) were retrieved (No data found for COL5A2 and COL3A1 was not detected in most of the samples) (Additional file 4: Fig. S4A–D).

### The collagen gene expressions were correlated with stromal and immune cell infiltration in glioma

**Fig. 4:**
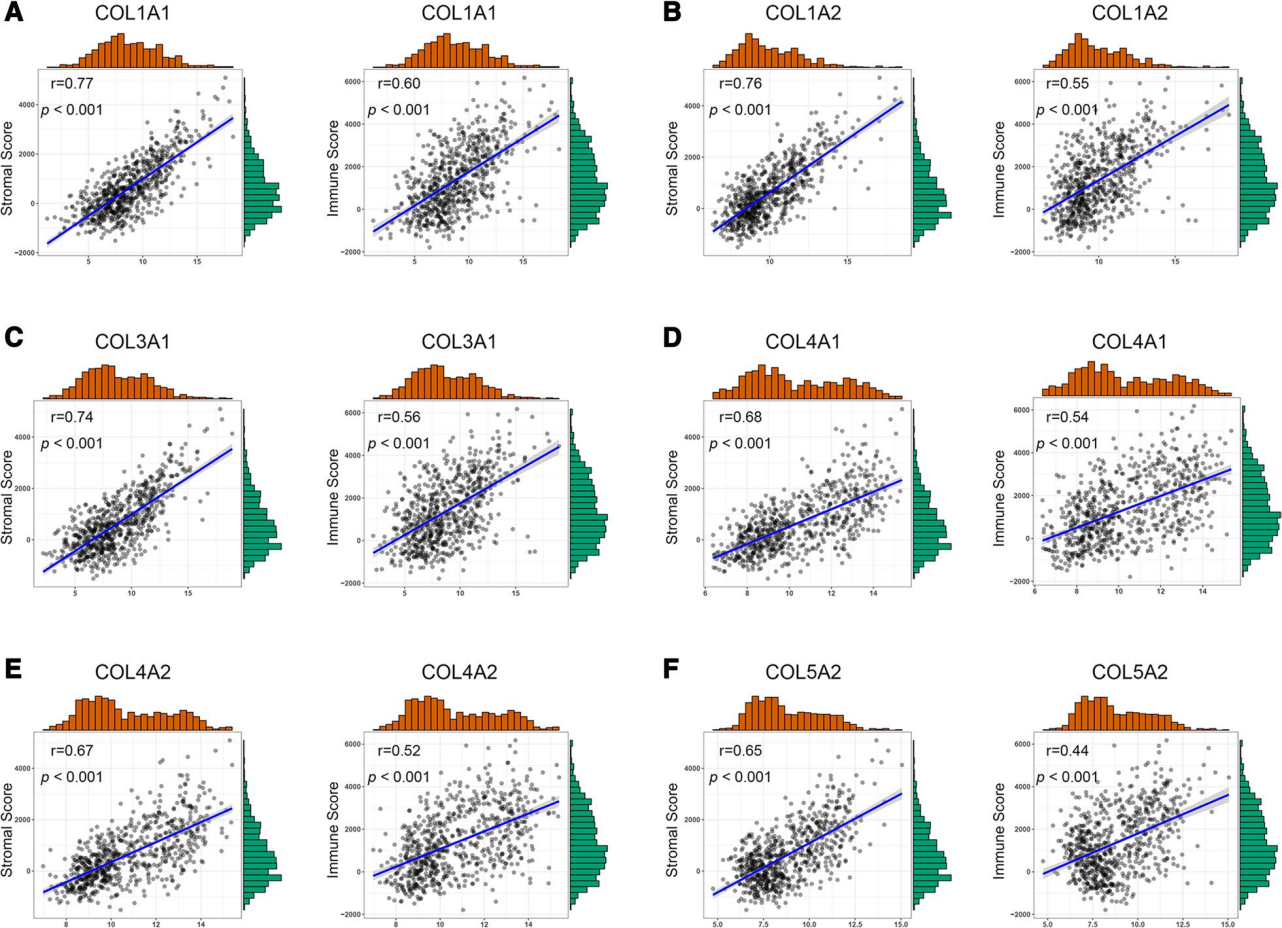
The collagen gene expressions were positively correlated with immunosuppressive properties. **A** Correlation between the collagen genes and immunosuppressive cell recruitment factors in TCGA dataset. **B** Correlation between the collagen genes and immunosuppressive factors in TCGA dataset. **C** Correlation between the collagen genes and immunosuppressive cell recruitment factors in the CGGA dataset. **D** Correlation between the collagen genes and immunosuppressive factors in the CGGA dataset. **E** Protein-protein interaction network of the collagen genes and immunosuppressive cell recruitment factors and immunosuppressive factors

The tumor microenvironment consists of various immune and stromal cells, which have been considered to closely correlate with patient prognosis. The correlations between collagen gene expressions and ESTIMATE scores were examined. Results showed that collagen gene expressions were significantly positively correlated with stromal and immune scores in TCGA dataset (Fig. 4A–F). Moreover, collagen genes also showed a significant correlation with stromal and immune scores in GEO and CGGA datasets (Additional file 5: Fig. 5A-B).

### The collagen gene expressions were correlated with immunosuppressive properties in glioma

**Fig. 5:**
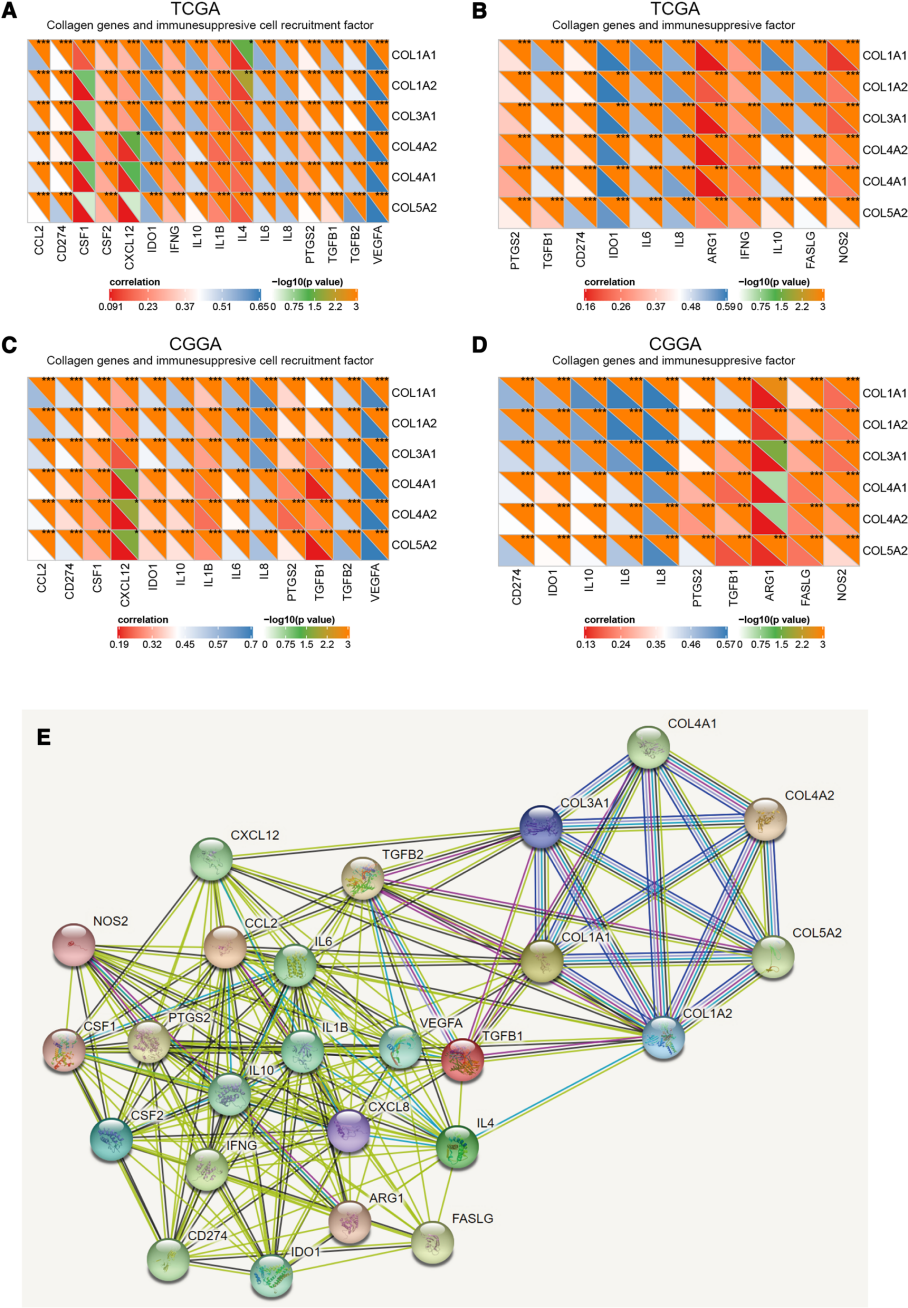
The collagen genes were positively correlated with significantly involved in the EMT process of Glioma. **A**-**C** Correlation between the collagen genes and biomarkers of epithelial-mesenchymal transition (EMT). **D** Protein-protein interaction network of the collagen genes and EMT-related genes

Stromal and immune cells can secrete many factors that cultivate a chronic inflammatory and immunosuppressive intratumoral atmosphere. We found that the collagen genes were significantly positively correlated with the majority of immunosuppressive and immunosuppressive cell recruitment factors (Fig. 5A–D). Moreover, a PPI network of collagen genes and immunosuppressive and immunosuppressive cell recruitment factors showed that they were closely correlated with each other (Fig. 5E). To validate the above findings, we selected the COL1A1 and IL-6 for further study and detected whether they were co-expression in glioma tissues. IL-6, as an important immunosuppressive and immunosuppressive cell recruitment factors, has a profound effect on immune cell infiltration in the tumor immune microenvironment [28, 29]. The double-immunofluorescence staining revealed that the co-expression of COL1A1 with IL-6 was found in WHO II-IV grade glioma tissues (Additional file 6: Fig. S6).

### The collagen gene expressions were closely associated with immune cell infiltration in glioma

**Fig. 6:**
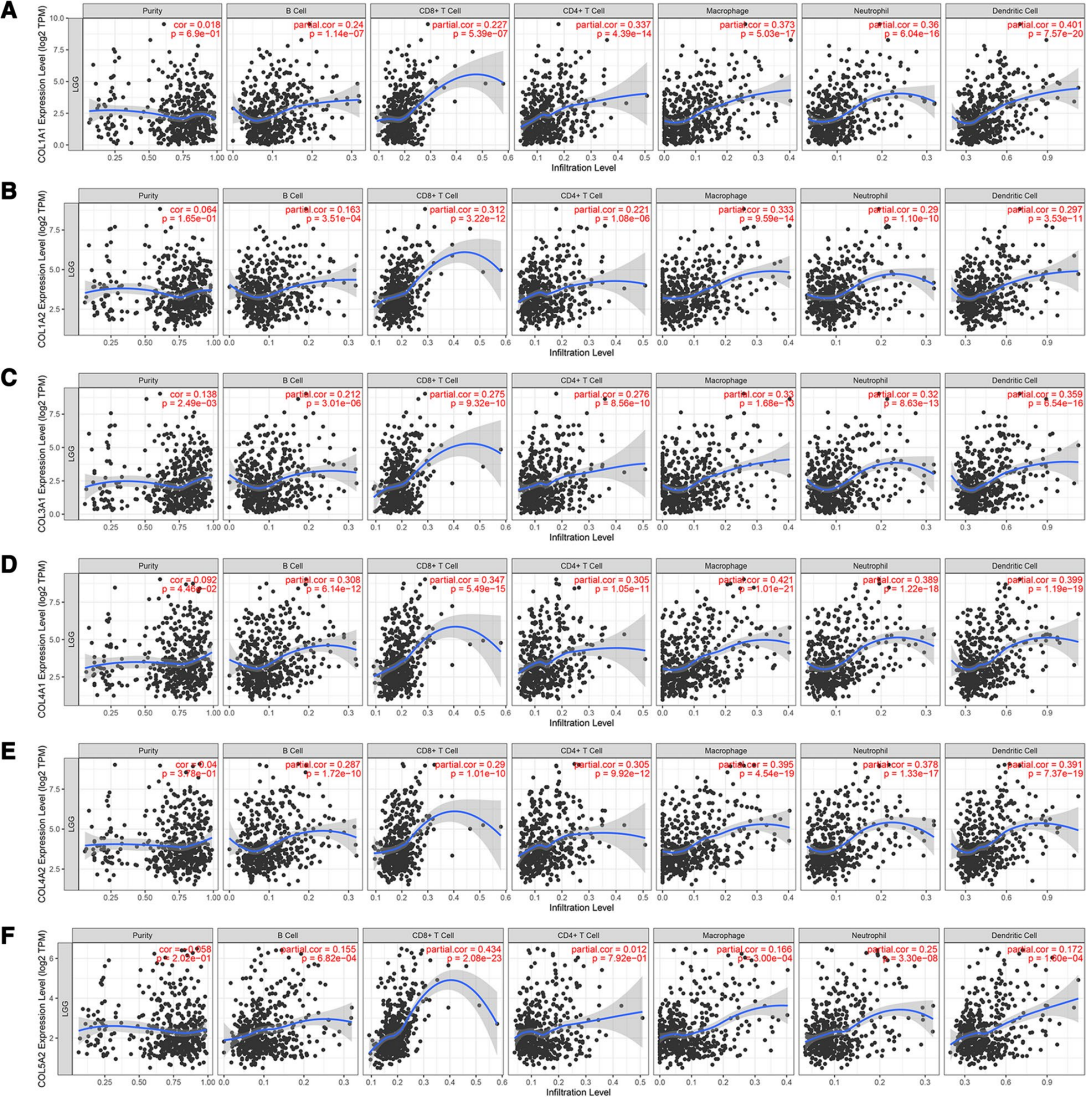
The correlation between the collagen genes and immune cell infiltration (TIMER) in LGGs. **A** COL1A1, **B** COL1A2, **C** COL3A1, **D** COL4A1, **E** COL4A2, and **F** COL5A2. *p*-value < 0.05 represented statistically significant

We comprehensively explored the correlation between the collagen genes and immune cell infiltration in both LGGs and GBM by using the TIMER database. The results revealed that there was a positive correlation between the expression of COL1A1, COL1A2, COL3A1, COL4A1, and COL4A2, and the infiltration of B cells, CD8 + T cells, CD4 + T cells, macrophages, neutrophils, and dendritic cells in LGGs (*p* < 0.05) (Fig. 6A–E). Except for in CD4 + T cells, the COL5A2 expression was positively correlated with the infiltration of the other five immune cell types (B cells, CD8 + T cells, macrophages, neutrophils, and dendritic cells; all *p* < 0.05) in LGGs (Fig. 6F). Besides, the expression levels of 6 collagen genes were all positively correlated with the infiltration of dendritic cells (all *p* < 0.05) in GBM (Additional file 7:  Fig. S7A–F).

### The collagen genes were significantly involved in the EMT process of glioma

**Fig. 7:**
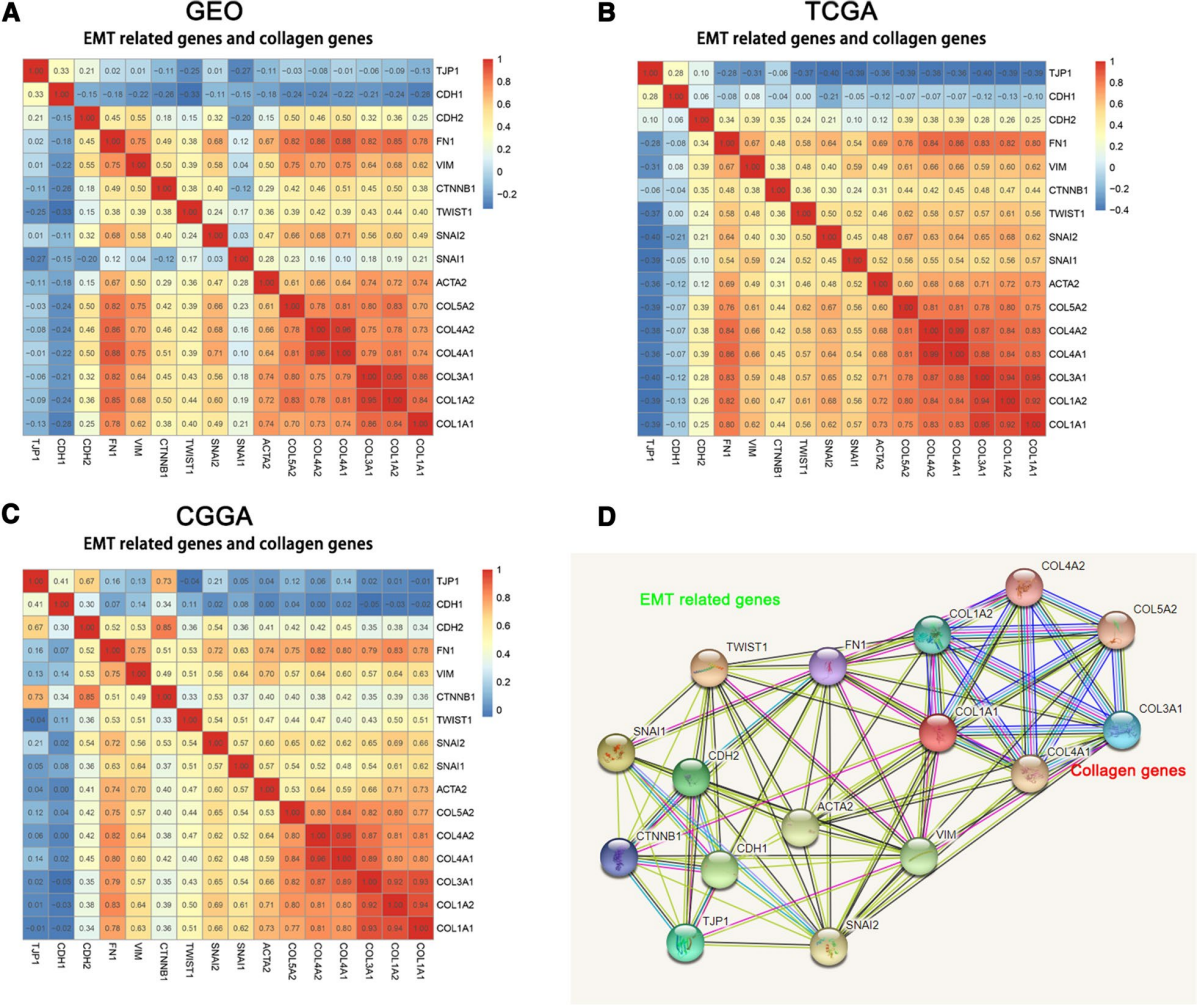
The collagen genes were significantly involved in the EMT process of Glioma. **A**–**C** Correlation between the collagen genes and biomarkers of epithelial-mesenchymal transition (EMT). **D** Protein-protein interaction network of the collagen genes and EMT-related genes

FN1, VIM, SNAI2, ACTA2, CTNNB1, TWIST1, and SNAI1 are important EMT biomarkers [22, 30]. As shown in Fig. 7 A, they were strongly or moderately positively correlated with the collagen genes (COL1A1, COL1A2, COL3A1, COL4A1, COL4A2, and COL5A2) based on the TCGA dataset. Moreover, we conducted a PPI network analysis of collagen genes and EMT-related genes to explore the potential interactions among them. As expected, these collagen genes were closely correlated with the EMT-related genes (Fig. 7B).

### Knockdown of COL3A1 suppressed migration, invasion and EMT process in glioma cells

**Fig. 8:**
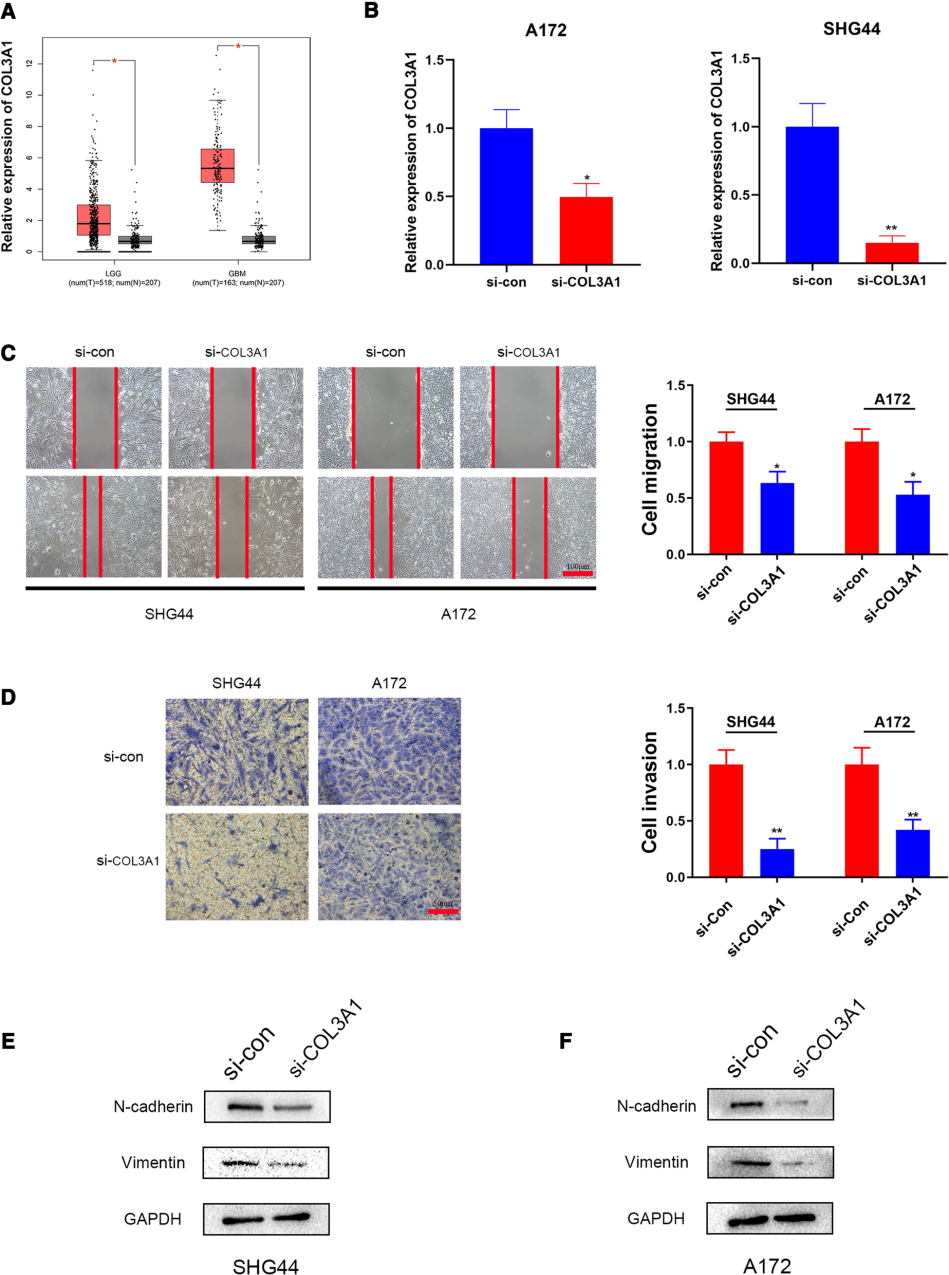
Knockdown of COL3A1 inhibiting migration, invasion, and EMT process. **A** Relative expression of COL3A1 in glioma (LGG and GBM) and normal brain tissue. After COL3A1 siRNA transfected, SHG44 and A172 (**B**) cells showed reduced COL3A1 levels measured by qRT-PCR. **C** Wound healing assays indicated that the migratory ability of glioma cells was reduced after transfection. **D** Transwell assays revealed that the invasiveness ability of glioma cells was inhibited after transfection. **E**-**F** The protein levels of Vimentin and N-cadherin were detected by Western blotting analysis in SHG44 and A172 cells after COL3A1 knockdown with siRNA. Data represented as mean value ± SD. * *p* < 0.05, ** *p* < 0.01

To further validate the collagen genes were involved in the EMT process, we took COL3A1 for the subsequent analysis. Based on the GEPIA database, the expression of COL3A1 in glioma (LGG and GBM) was higher than normal samples (Fig. 8A). To further confirm the biological function of COL3A1 on glioma progress, COL3A1 was knockdown by using small interfering RNA (siRNA) in SHG44 and A172 cells. After transfected with siCOL3A1, the expression of COL3A1 was significantly down-regulated in SHG44 and A172 cells (Fig. 8B). On account of the EMT program usually accompanied by the changes in cell migration and invasion, then, wound healing and transwell assays were conducted to detect the migratory and invasive ability of glioma cells. The result showed that knockdown of COL3A1 leads to delayed wound healing and decreased invasive ability (Fig. 8C–D). Considering the E-cadherin was poorly expressed in glioma cells, we tested the expression of two common EMT markers (Vimentin and N-cadherin) to analyze the EMT process of glioma. As expected, the protein levels of Vimentin and N-cadherin were significantly down-regulated after the reduction of COL3A1 in SHG44 and A172 cells (Fig. 8E–F). These findings indicated that COL3A1 promotes migration, invasion, and EMT process in glioma.

## Discussion

Whenever possible, the current standard therapy of malignant glioma is surgical resection [3], which could achieve cytoreduction, relieve mass effect and provide adequate tissue for tumor histologic characterization and establishing molecular genotype [31]. Combined radiotherapy, chemotherapy, and chemoradiation therapy is also needed, but it is far from combating the tumor progression [32]. A more comprehensively prognostic classification integrating genetic and epigenetic signatures is even more important than clinical factors. This integrated pattern may be more accurate in clinical diagnosis and management decisions. Despite a deeper understanding of molecular and genetic diversity and improvements in therapeutic strategies, the current treatment effect for the majority of patients, including concomitant chemotherapy and biologically targeted agents, remain disappointing [33]. Thus, molecular characterization based on glioma progression is urgently needed for further elucidating novel and essential information, understanding underlying aggression mechanisms, and providing prospective prognostic and therapeutic biomarkers.

WGCNA is a robust method of constructing gene co-expression networks to illustrate gene expression profiles, detect modules based on similarities of expression features and recognize candidate module and hub genes related to clinical information. It has been successfully applied in a variety of tumors to explore potential crucial genes of clinical significance [34, 35]. In the present study, we identified 4219 DEGs by using bioinformatics analysis in the public microarray datasets (GSE43378 and GSE7696) between glioma samples and non-tumor samples. These DEGs were divided into 7 modules according to intramodule connectivity and the yellow module was selected for further exploring because of its most prominent correlation with clinical features. Functional and enrichment analyses of the yellow module may provide a novel viewpoint to illuminate the mechanism of glioma progression. GO annotation analysis revealed that genes in the yellow module were mainly focused on ECM, indicating that ECM was likely to play crucial roles in the progression of glioma. Moreover, biological pathways demonstrated that KEGG was significantly focused on ECM-receptor interaction. These findings may help to explain the mechanism of how it influenced the progression and survival of glioma. were consistent with previous studies.

Collagens are the most significant protein component of the ECM [8]. Increased deposition of collagen alone or in combination facilitates cancer cell proliferation, migration, and metastasis by interfering with cell polarity, cell-cell adhesion and ultimately resulting in amplification of the growth factor signaling [36]. COL1A1, collagen type I alpha 1 chain, has been identified as an invasion‑related gene in malignant astrocytomas [37]. COL1A2 was found to significantly promote gastric cancer cell proliferation, migration, apoptosis, and invasion [38]. In a recent study, the collagen molecules COL4A1 [39] and COL3A1 [40] were proved to be essential for the development of glioma. COL4A2 was significantly higher expressed in GBM than astrocytoma, which was confirmed by RT-qPCR [41]. COL5A2 has not reported the roles in glioma progression, but they were reported to participate in various malignancies. COL5A2 was involved in osteosarcoma cell proliferation and invasion, provided new insights into cytostatic drug resistance of ovarian cancer, and represented early potential diagnostic biomarkers and therapeutic targets for bladder cancer [42–44].

Although a previous study has been found collagen genes were related to the progression and prognosis of LGGs [45], no study has been focused on the correlation between collagen genes and the tumor microenvironment of glioma. The tumor microenvironment consists of inflammatory cells, stromal cells, fibroblasts, vascular endothelial cells, and ECM [46]. Collagens are important components of the ECM and play a critical role in the tumor microenvironment. In our study, the immune and stromal scores of each sample were calculated based on the ESTIMATE algorithm, and then the correlation between collagen genes and immune and stromal scores was analyzed. The result showed that collagen genes were significantly associated with immune and stromal scores. These findings confirmed that the collagen genes were significantly correlated with the immune microenvironment of glioma. Subsequent analysis showed that collagen genes were significantly positively correlated with immunosuppressive and immunosuppressive cell recruitment factors. Thus, collagen genes might be involved in regulating the immunosuppressive microenvironment of glioma. Immune cell infiltration could affect the progression and recurrence of glioma, and act as a significant determinant of prognosis and immunotherapy [47]. Based TIMER database, it was easily found that the infiltration levels of B cells, CD8 + T cells, CD4 + T cells, macrophages, neutrophils, and dendritic cells were significantly negatively correlated with the OS of LGGs, and the infiltration level of dendritic cells was significantly negatively correlated with the OS of GBM. In this study, we revealed that a significant positive correlation between the expression of the collagen genes and the infiltration of the six immune cell types (B cells, CD8 + T cells, CD4 + T cells, macrophages, neutrophils, and dendritic cells) in LGGs, and a positive correlation between the expression of the collagen genes and the infiltration of dendritic cells in GBM. That indicated that the collagen genes could not only serve as prognostic biomarkers but also reflect the immune status of glioma.

Collagen is a major ECM component in the stroma of many solid tumors. Cancers with excessive ECM lead to immunity resistance because it is a physical barrier between tumor cells and immune effectors [48]. This is supported by the fact that thickened ECM surrounding cancer cells and excessive ECM deposition resistance against immune checkpoint inhibitors [49, 50]. Additionally, collagen can regulate immune cell motility, metabolism, and survival. Loose, well-aligned collagen fibers can provide the optimal conditions for T-cell migration [48]. On the contrary, excessive collagen can promote immune cell infiltration but inhibit their anti-tumor activity in the tumor microenvironment [51, 52], which was consistent with our findings. A previous study also showed that high expression of COL1A1, COL3A1, COL5A1, and COL5A2 in ovarian cancer promotes tumor immune tolerance and results in poor prognosis [53]. Besides, overexpression of immunosuppressive molecules and overrepresentation of immunosuppressive cells (e.g. regulatory T cells, myeloid-derived suppressor cells, and tumor-associated macrophages) are the important immunosuppressive mechanisms in the tumor microenvironment [54]. In this study, PPI network analysis showed that collagen and immunosuppressive and immunosuppressive cell recruitment factors may have close interaction. More importantly, double-immunofluorescence staining demonstrated the co-expression of collagen and immunosuppressive and immunosuppressive cell recruitment factors in glioma samples. So, we supposed that immunosuppressive molecules, immunosuppressive cells, and collagen interact with each other in the tumor microenvironment of glioma, where they may maintain the immunosuppressive microenvironment together.

EMT, as a key factor of malignant glioma invasion enhancement, can be characterized by an increase in the expression of biomarkers, such as CDH2, VIM, FN1, SNAI1, SNAI2, ACTA2, and so on [55]. We found that collagen genes were positively correlated with EMT biomarkers, thus possibly involved in the EMT process of glioma. Furthermore, PPI analysis showed that EMT-related genes closely interact with the collagen genes. To further explore the function of collagen genes in promoting glioma progression, we used rarely studied COL3A1 as a further research object. The results showed that the knockdown of COL3A1 decreased the migration, invasion, and EMT process of glioma cells. Thus, we believe that the collagen genes are important EMT mediators in glioma (Additional file 8).

## Conclusions

In conclusion, the collagen genes (COL1A1, COL1A2, COL3A1, COL4A1, COL4A2, and COL5A2) could regulate the immunosuppressive microenvironment and be involved in the EMT process of glioma. Thus, collagen genes participate in the malignant progression of glioma and could serve as potential therapeutic targets for glioma management.

## Supplementary Information

**Additional file 1: Fig. S1.** The most significantly enriched GO terms and KEGG pathways of the yellow module; and the weighted co-expression network of 6 collagen genes (COL1A1, COL1A2, COL3A1, COL4A1, COL4A2, and COL5A2). **A** Top 6 significantly enriched BP annotations; **B** top 6 significantly enriched MF annotations; **C** top 6 significantly enriched CC annotations; **D** top 6 significantly enriched KEGG pathways. The length of bars reflects the number of genes, and colors reflect the p-value. **E** Weighted co-expression network of collagen genes in the yellow module, which was visualized in Cytoscape. Red and blue nodes stand for the collagen genes and other co-expressed genes.

**Additional file 2: Fig. S2.** mRNA levels of collagen genes in glioma (ONCOMINE). **A** The expression level of collagen genes in various cancers. **B** COL1A1, COL1A2, COL3A1, COL4A1, COL4A2, and COL5A2 mRNA levels were positively correlated with WHO grades based on the Sun Brain dataset from Oncomine. Different colors represent different grades (blue represented unassigned value).

**Additional file 3: Fig. S3.** Glioma grade plots of the collagen genes in the CGGA database. COL1A1 (**A**), COL1A2 (**B**), COL3A1 (**C**), COL4A1 (**D**), COL4A2 (**E**), and COL5A2 (**F**) mRNA levels were positively correlated with WHO grades based on the CGGA database. * p<0.05, ** p<0.01, *** p<0.001 or **** p<0.0001.

**Additional file 4: Fig. S4.** The protein-level of collagen genes in the Human Protein Atlas database (immunohistochemistry). The translational expression level of the 4 collagen genes was positively correlated with WHO grade in glioma samples. **A** COL1A1, **B** COL1A2, **C** COL4A1, **D** COL4A2. No data found for COL5A2 and COL3A1 was not detected in most of the samples.

**Additional file 5: Fig. S5.** The collagen gene expressions were positively correlated with stromal, immune score, and ESTIMATE scores in glioma patients. **A** GEO dataset, **B** CGGA dataset.

**Additional file 6: Fig. S6.** Co-expression of COL1A1 with IL6 in WHO II-IV grade glioma tissues using immunofluorescence.

**Additional file 7: Fig. S7.** The correlation between the collagen genes and immune cell infiltration (TIMER) in GBM. **A** COL1A1, **B** COL1A2, **C** COL3A1, **D** COL4A1, **E** COL4A2, and **F** COL5A2. p-value < 0.05 represented statistically significant.

**Additional file 8: Fig. S8.**. The original data of the experimental validation.

## Data Availability

The datasets used in the current study are included within the article. Other data and materials are available from the corresponding author on reasonable request.

## Abbreviations

BP: Biological process
CC: Cellular component
CGGA: Chinese Glioma Genome Atlas
CNS: Central nervous system
DEG: Differentially expressed gene
DAVID: Database for Annotation, Visualization, and Integrated Discovery
ECM: Extracellular matrix
EMT: Epithelial-mesenchymal transition
ESTIMATE: Estimation of STromal and Immune cells in MAlignant Tumors using Expression data
FDR: False discovery rate
GBM: Glioblastoma
GEO: Gene Expression Omnibus
GEPIA: Gene Expression Profiling Interactive Analysis
GO: Gene ontology
GS: Gene significance
KEGG: Kyoto Encyclopedia of Genes and Genomes
LGG: Lower-grade glioma
ME: Module eigengene
MF: Molecular function
MM: Module membership
OS: Overall survival
TCGA: The Cancer Genome Atlas
TIMER: Tumor Immune Estimation Resource
TOM: Topological overlap matrix
WGCNA: Weighted gene co-expression network analysis
WHO: World Health Organization
