# Supplementary figures and images for: Identification of collagen genes related to immune infiltration and epithelial-mesenchymal transition in glioma
Source: Cancer Cell Int. 2021 May 25;21:276. doi: 10.1186/s12935-021-01982-0 (PMC8147444; doi:10.1186/s12935-021-01982-0)

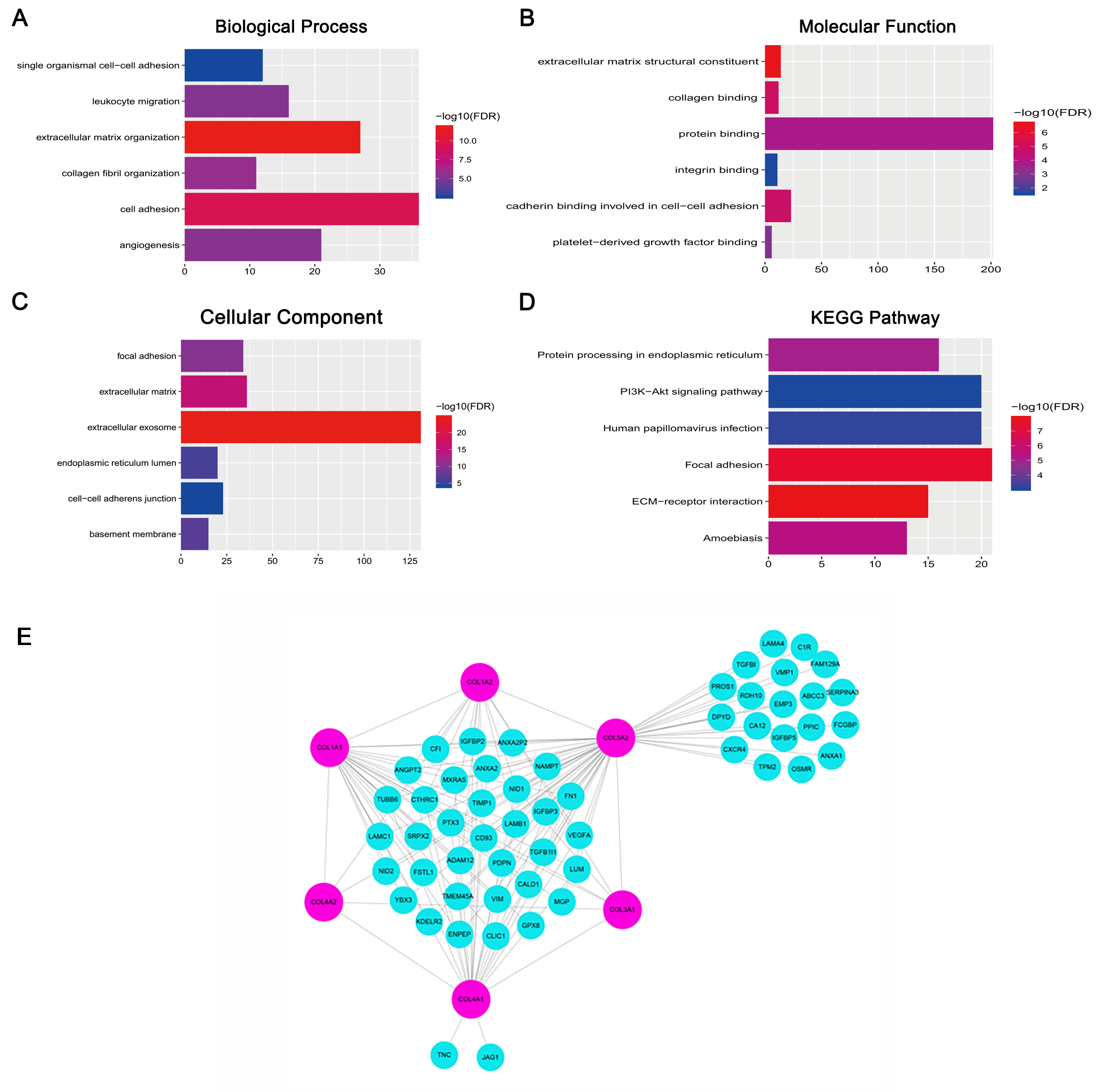

Supplement: Supplementary file 1 — Additional file 1: Fig. S1. The most significantly enriched GO terms and KEGG pathways of the yellow module; and the weighted co-expression network of 6 collagen genes (COL1A1, COL1A2, COL3A1, COL4A1, COL4A2, and COL5A2). A Top 6 significantly enriched BP annotations; B top 6 significantly enriched MF annotations; C top 6 significantly enriched CC annotations; D top 6 significantly enriched KEGG pathways. The length of bars reflects the number of genes, and colors reflect the p-value. E Weighted co-expression network of collagen genes in the yellow module, which was visualized in Cytoscape. Red and blue nodes stand for the collagen genes and other co-expressed genes. [file 12935_2021_1982_MOESM1_ESM.tif]

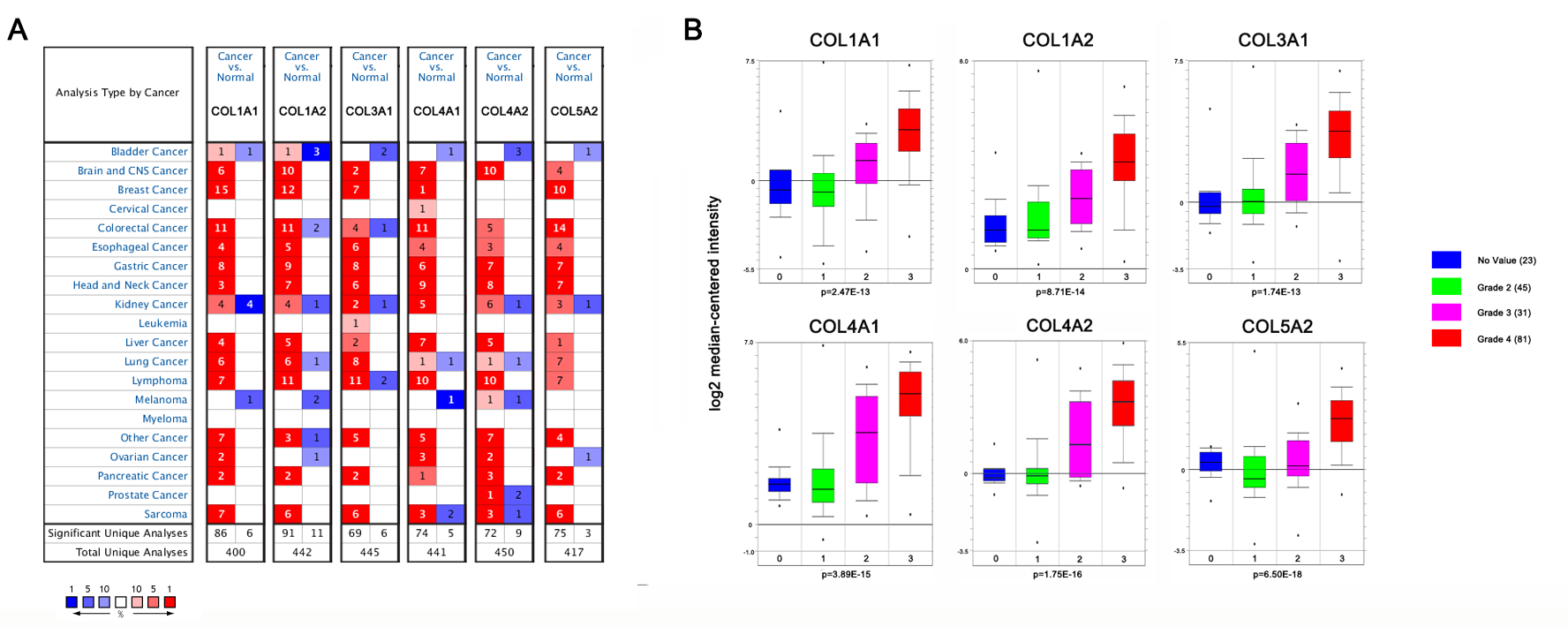

Supplement: Supplementary file 2 — Additional file 2: Fig. S2. mRNA levels of collagen genes in glioma (ONCOMINE). A The expression level of collagen genes in various cancers. B COL1A1, COL1A2, COL3A1, COL4A1, COL4A2, and COL5A2 mRNA levels were positively correlated with WHO grades based on the Sun Brain dataset from Oncomine. Different colors represent different grades (blue represented unassigned value). [file 12935_2021_1982_MOESM2_ESM.tif]

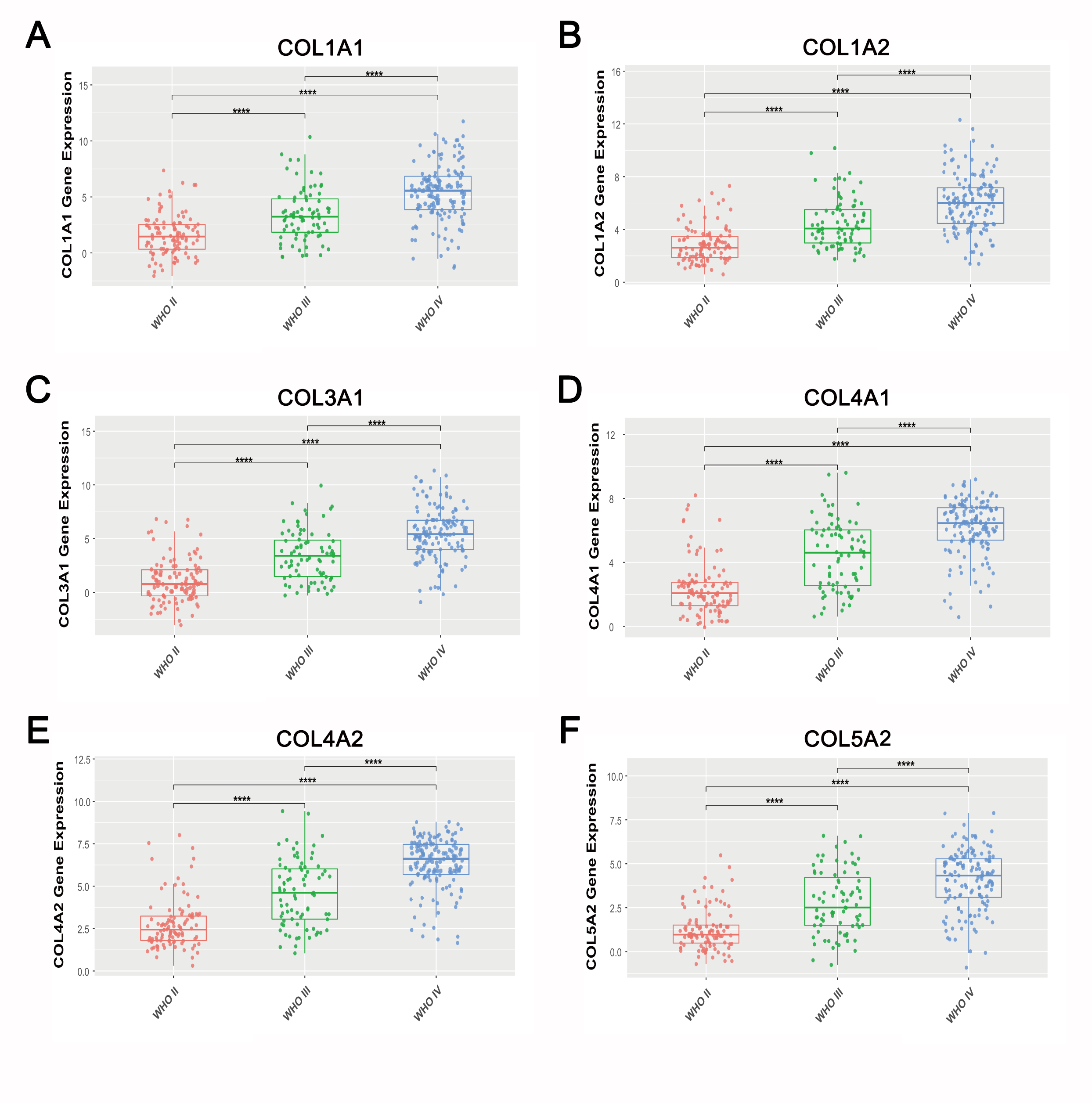

Supplement: Supplementary file 3 — Additional file 3: Fig. S3. Glioma grade plots of the collagen genes in the CGGA database. COL1A1 (A), COL1A2 (B), COL3A1 (C), COL4A1 (D), COL4A2 (E), and COL5A2 (F) mRNA levels were positively correlated with WHO grades based on the CGGA database. * p<0.05, ** p<0.01, *** p<0.001 or **** p<0.0001. [file 12935_2021_1982_MOESM3_ESM.tif]

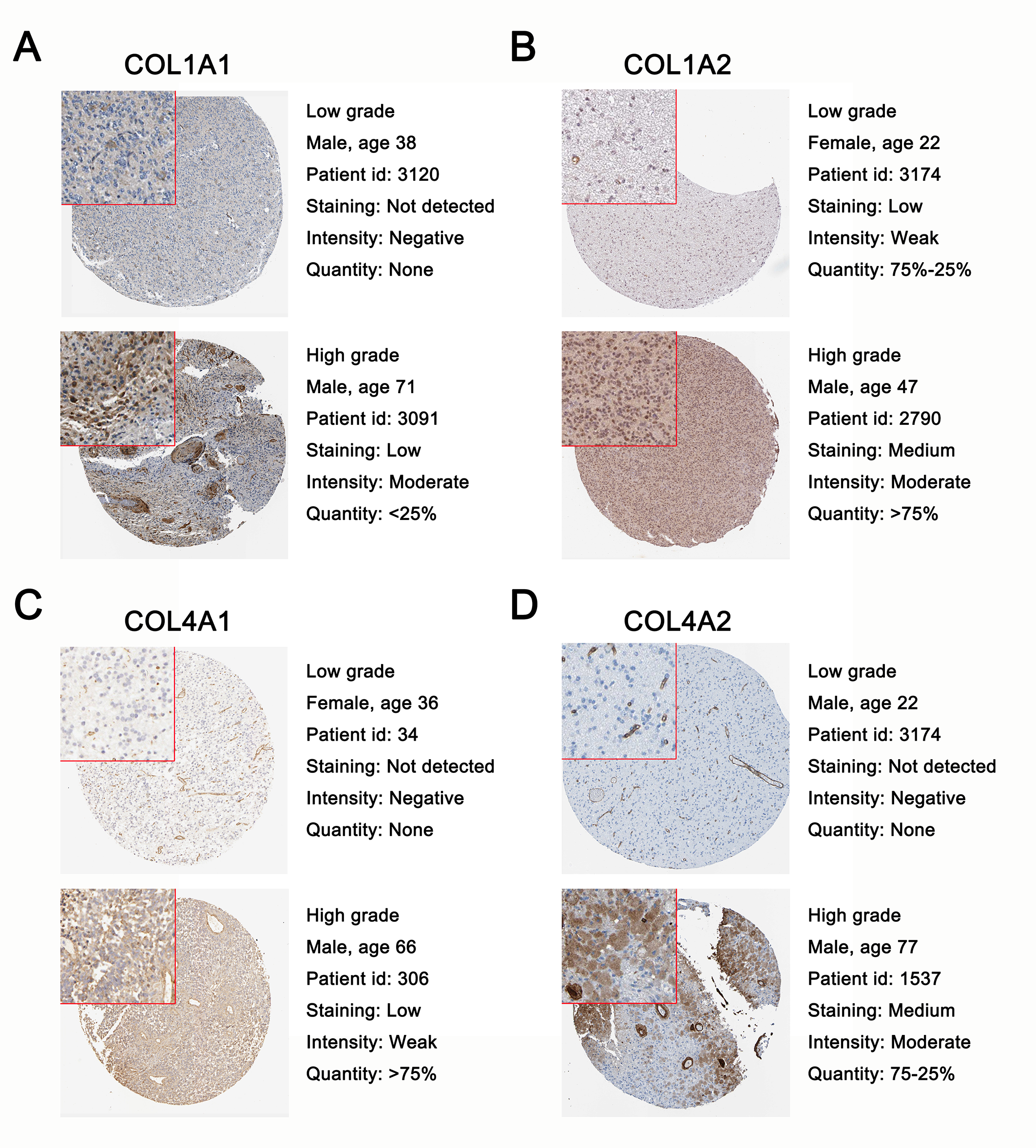

Supplement: Supplementary file 4 — Additional file 4: Fig. S4. The protein-level of collagen genes in the Human Protein Atlas database (immunohistochemistry). The translational expression level of the 4 collagen genes was positively correlated with WHO grade in glioma samples. A COL1A1, B COL1A2, C COL4A1, D COL4A2. No data found for COL5A2 and COL3A1 was not detected in most of the samples. [file 12935_2021_1982_MOESM4_ESM.tif]

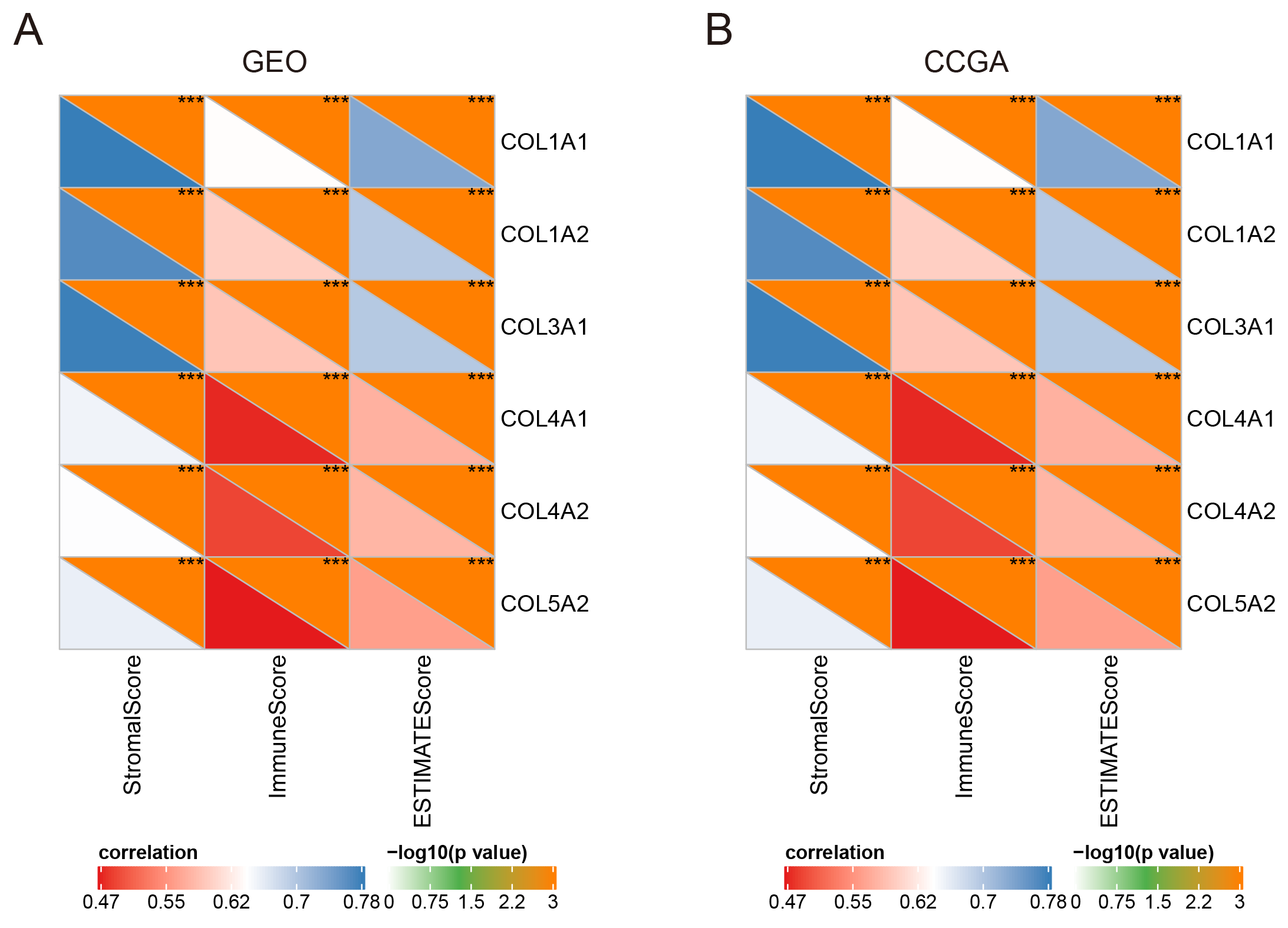

Supplement: Supplementary file 5 — Additional file 5: Fig. S5. The collagen gene expressions were positively correlated with stromal, immune score, and ESTIMATE scores in glioma patients. A GEO dataset, B CGGA dataset. [file 12935_2021_1982_MOESM5_ESM.tif]

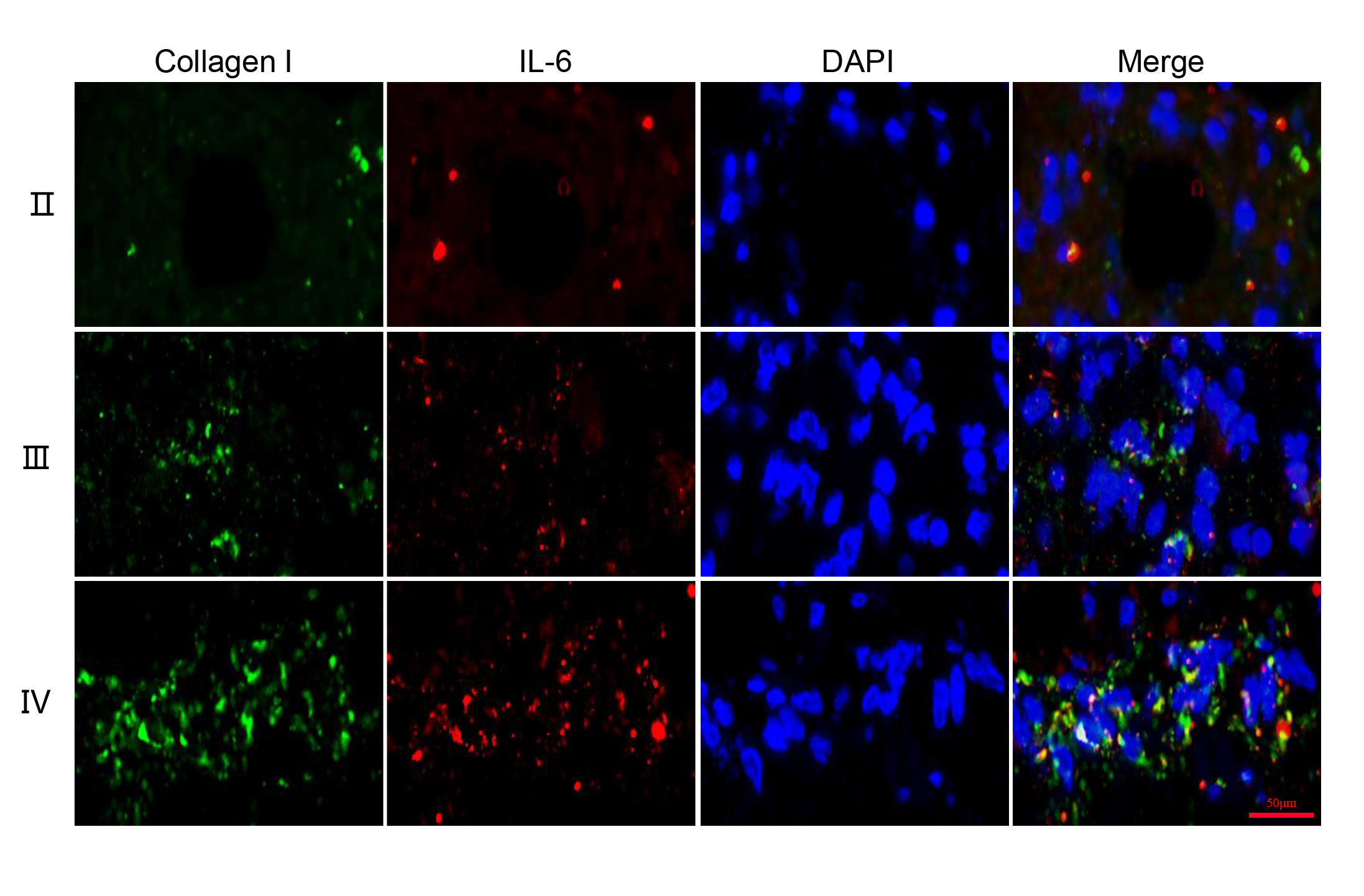

Supplement: Supplementary file 6 — Additional file 6: Fig. S6. Co-expression of COL1A1 with IL6 in WHO II-IV grade glioma tissues using immunofluorescence. [file 12935_2021_1982_MOESM6_ESM.tif]

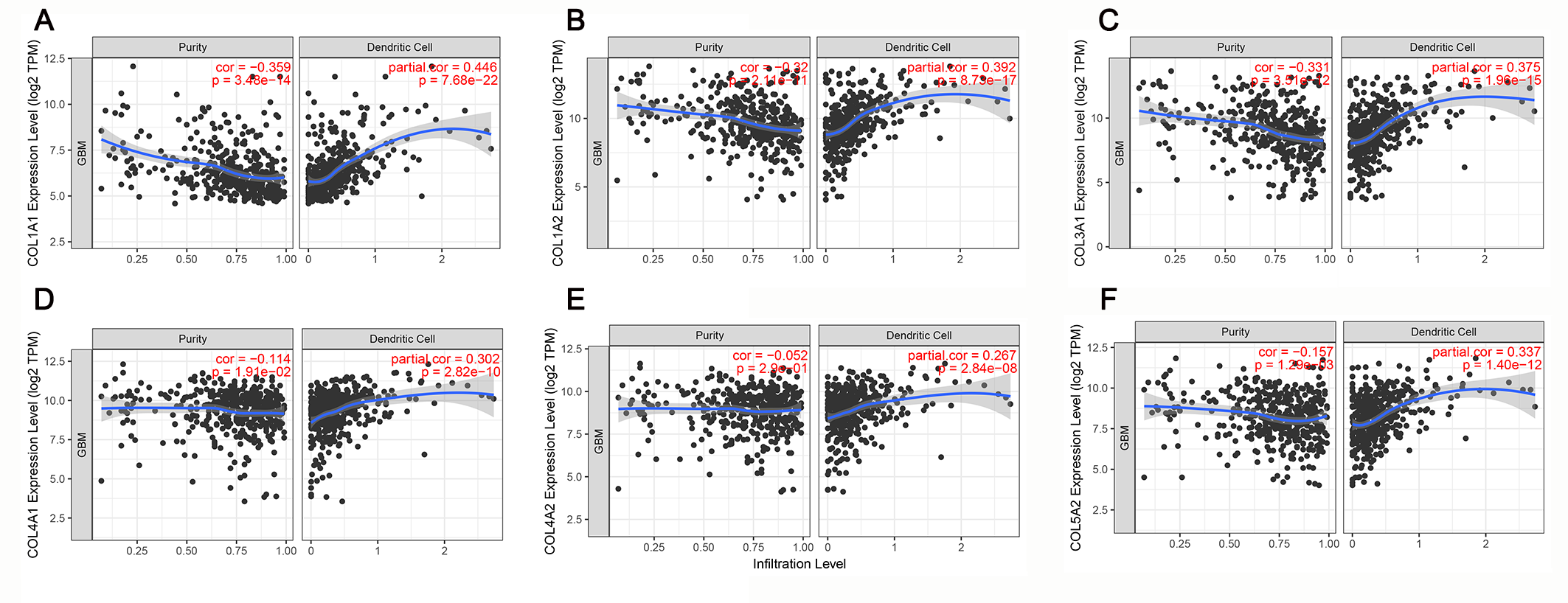

Supplement: Supplementary file 7 — Additional file 7: Fig. S7. The correlation between the collagen genes and immune cell infiltration (TIMER) in GBM. A COL1A1, B COL1A2, C COL3A1, D COL4A1, E COL4A2, and F COL5A2. p-value < 0.05 represented statistically significant. [file 12935_2021_1982_MOESM7_ESM.tif]
