# Supplementary material for: Identification of collagen genes related to immune infiltration and epithelial-mesenchymal transition in glioma
Source: Cancer Cell Int. 2021 May 25;21:276. doi: 10.1186/s12935-021-01982-0 (PMC8147444; doi:10.1186/s12935-021-01982-0)

Wound healing assay:

1. SHG44-NC-0h


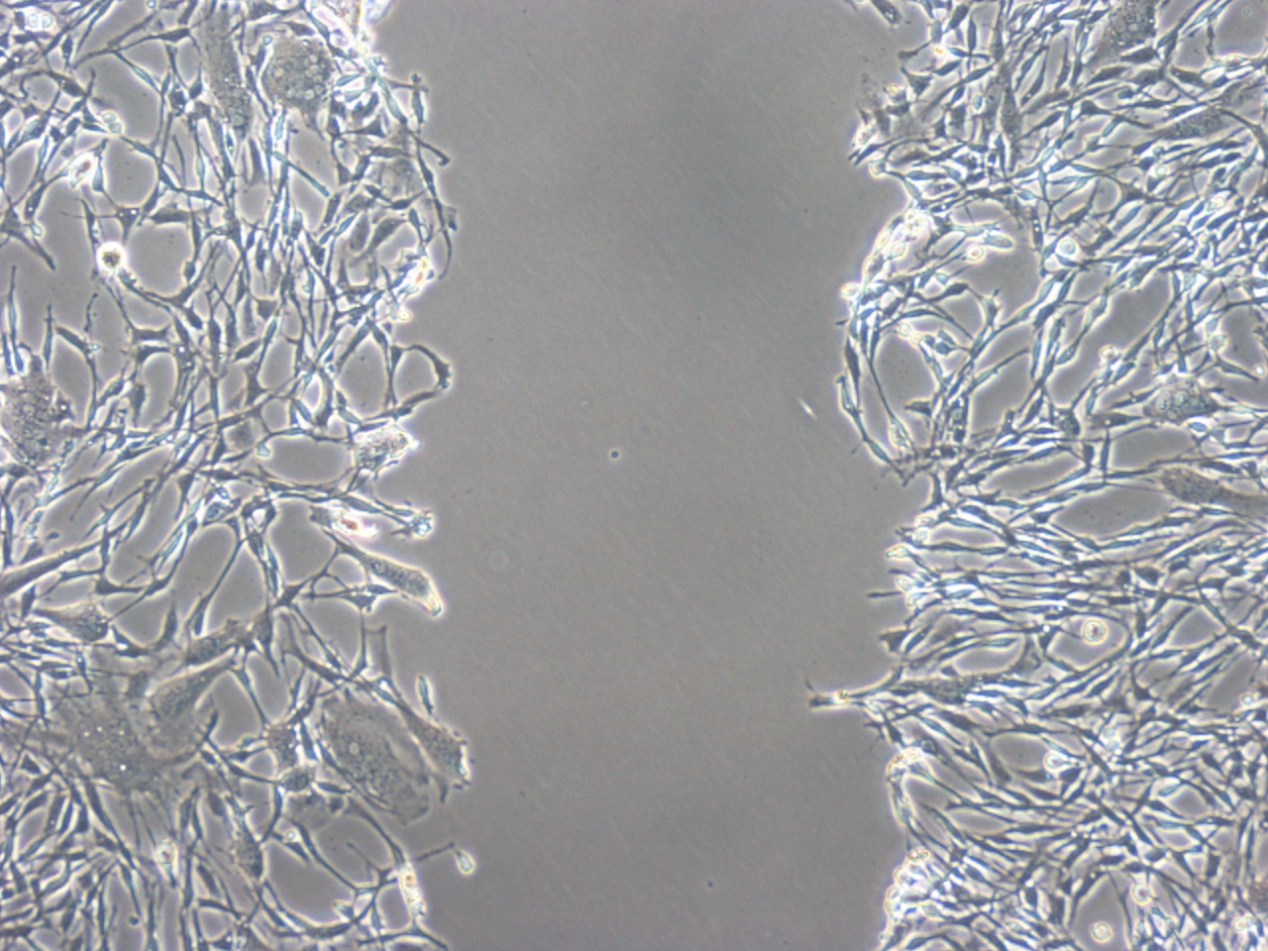


1. SHG44-NC-24h


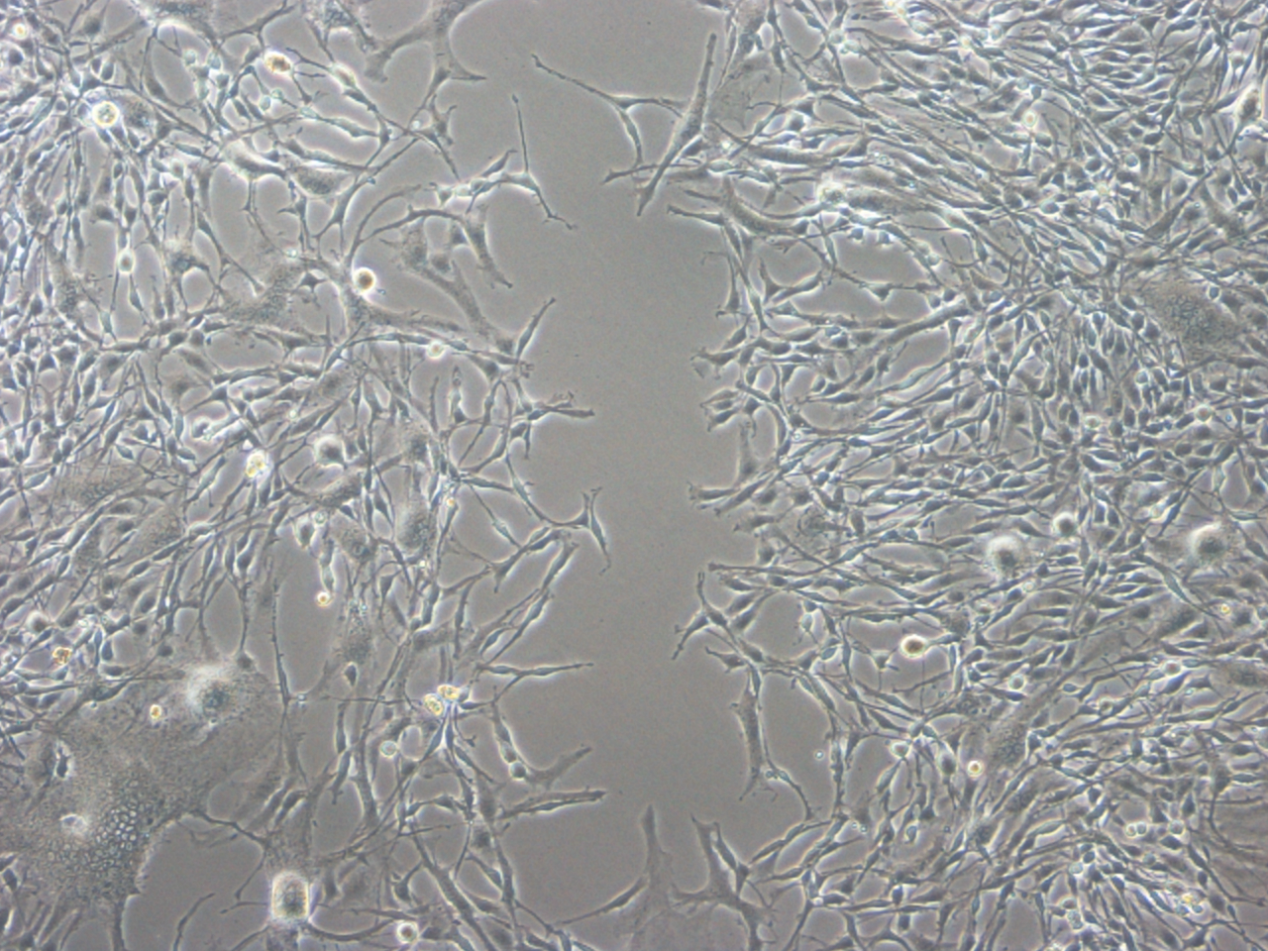


1. SHG44-si-COL3A1-0h


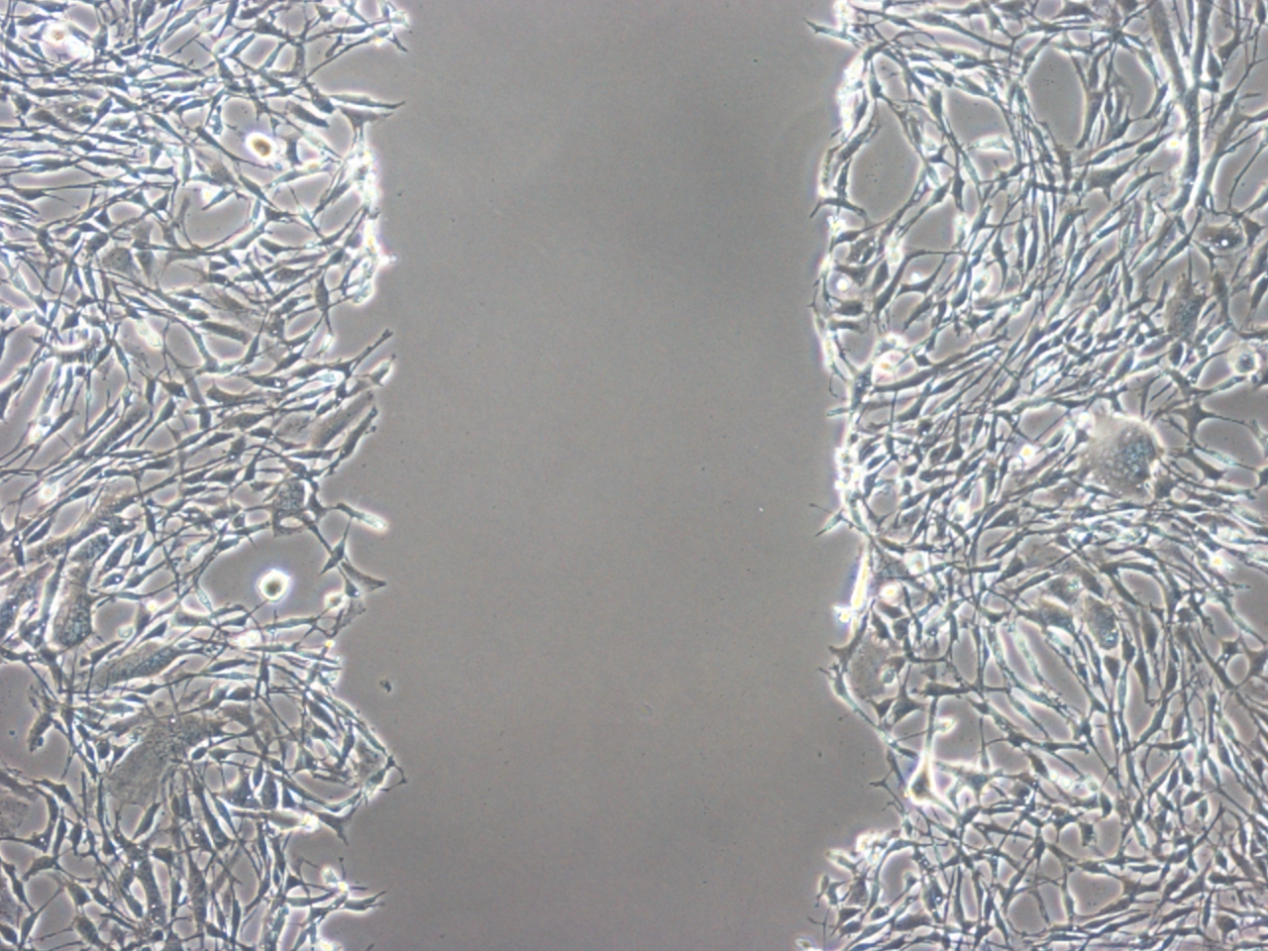


1. SHG44- si-COL3A1-24h


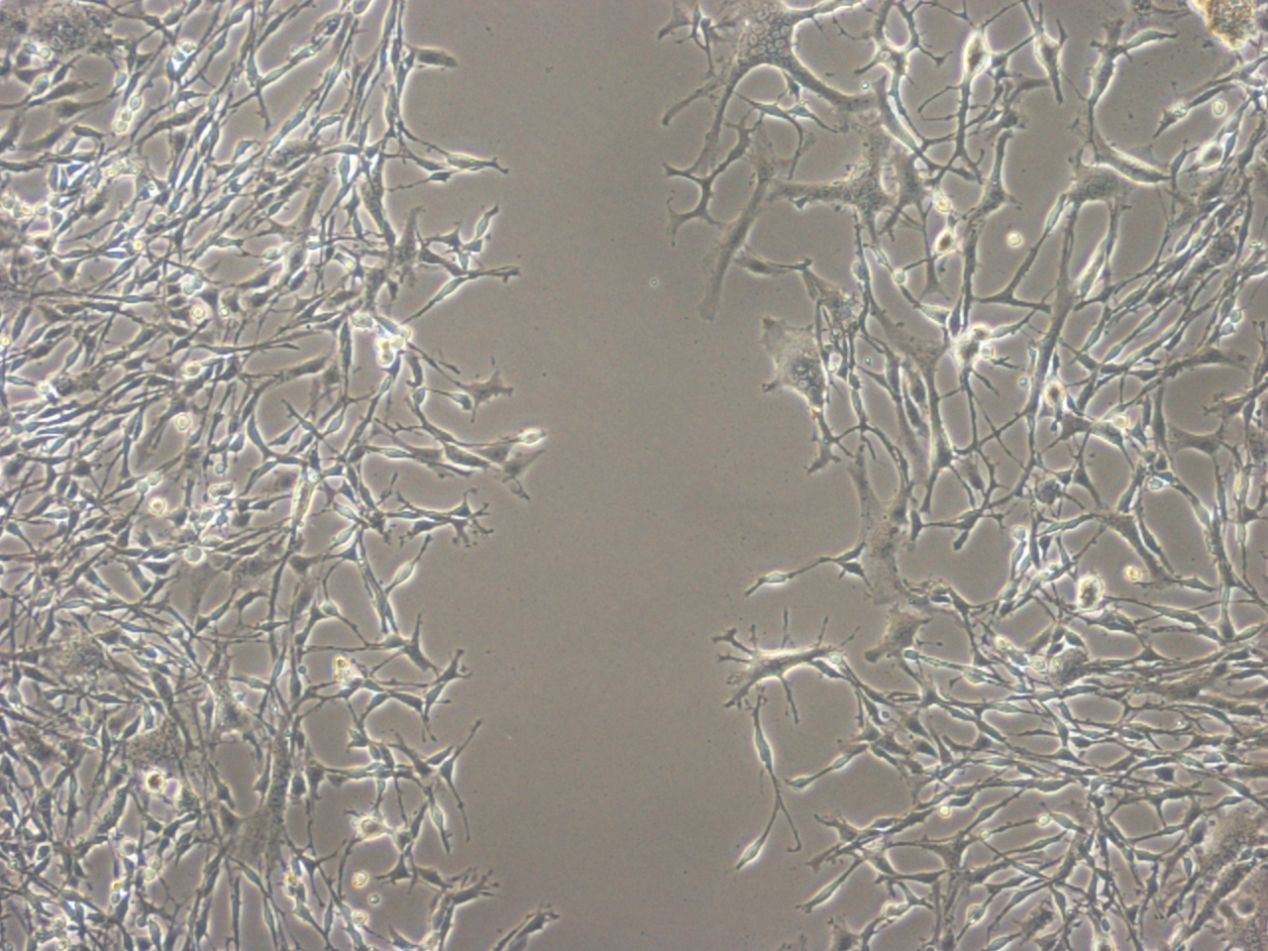


1. A172-NC-0h


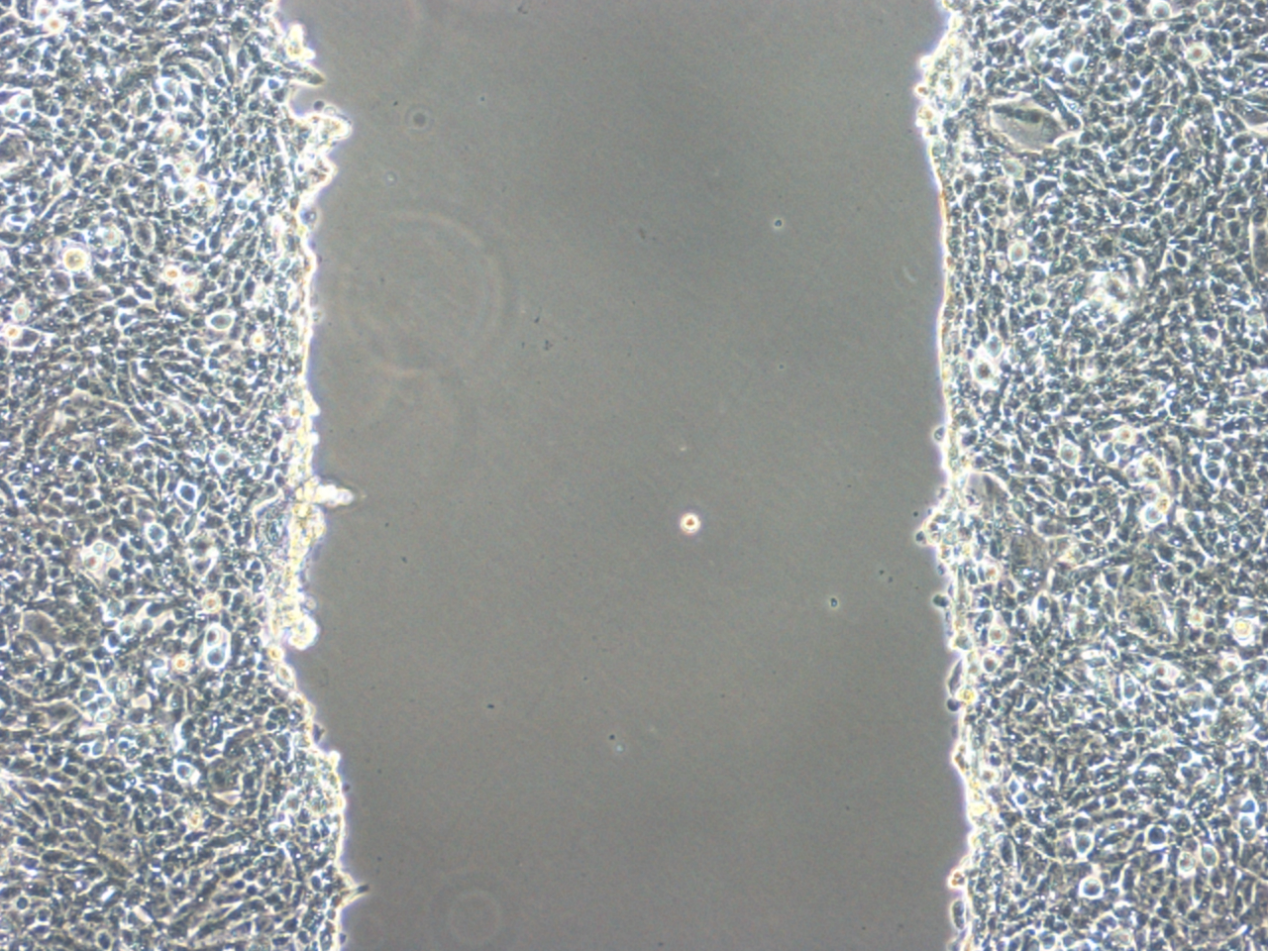


1. A172-NC-24h


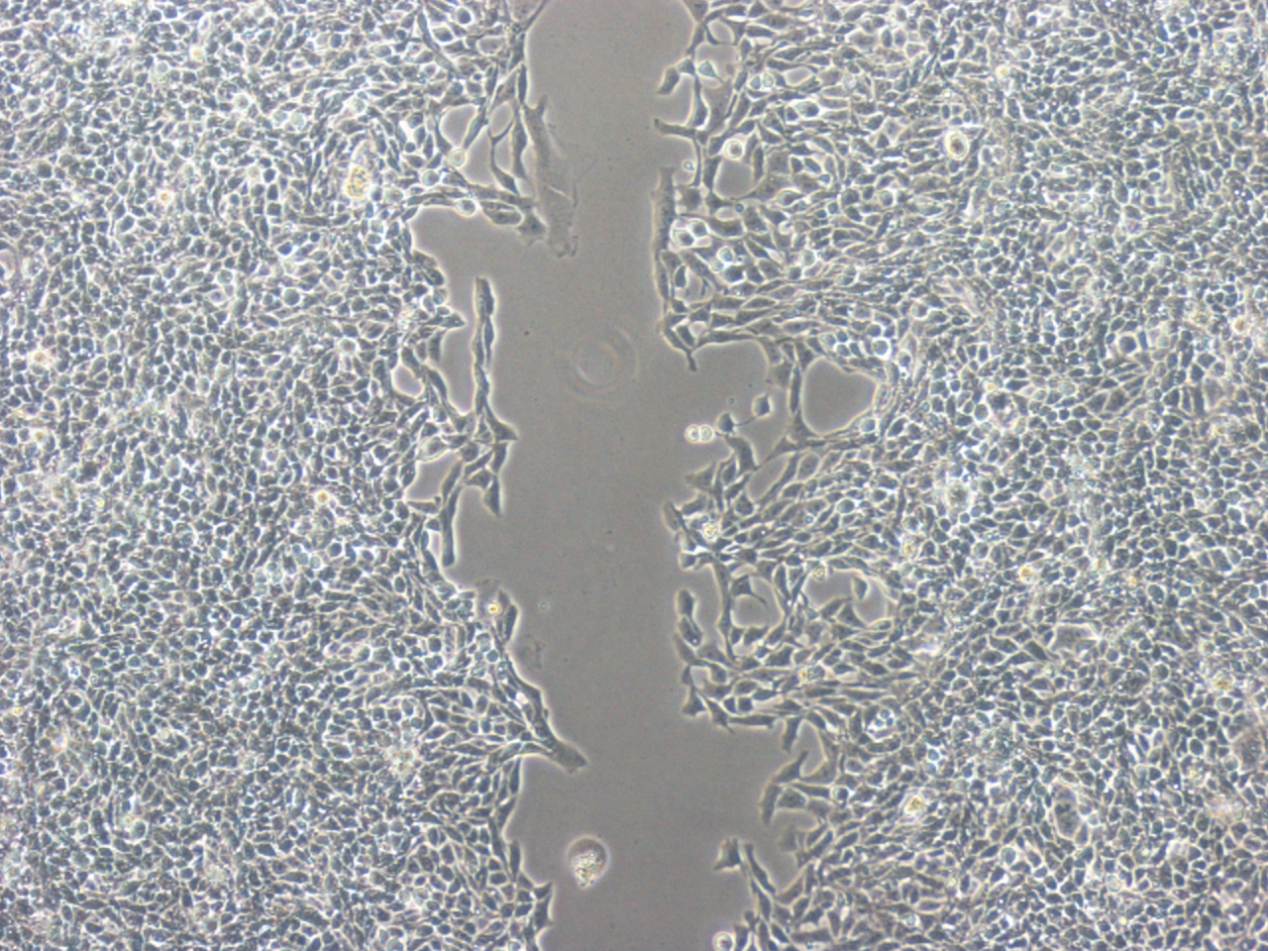


1. A172-si-COL3A1-0h


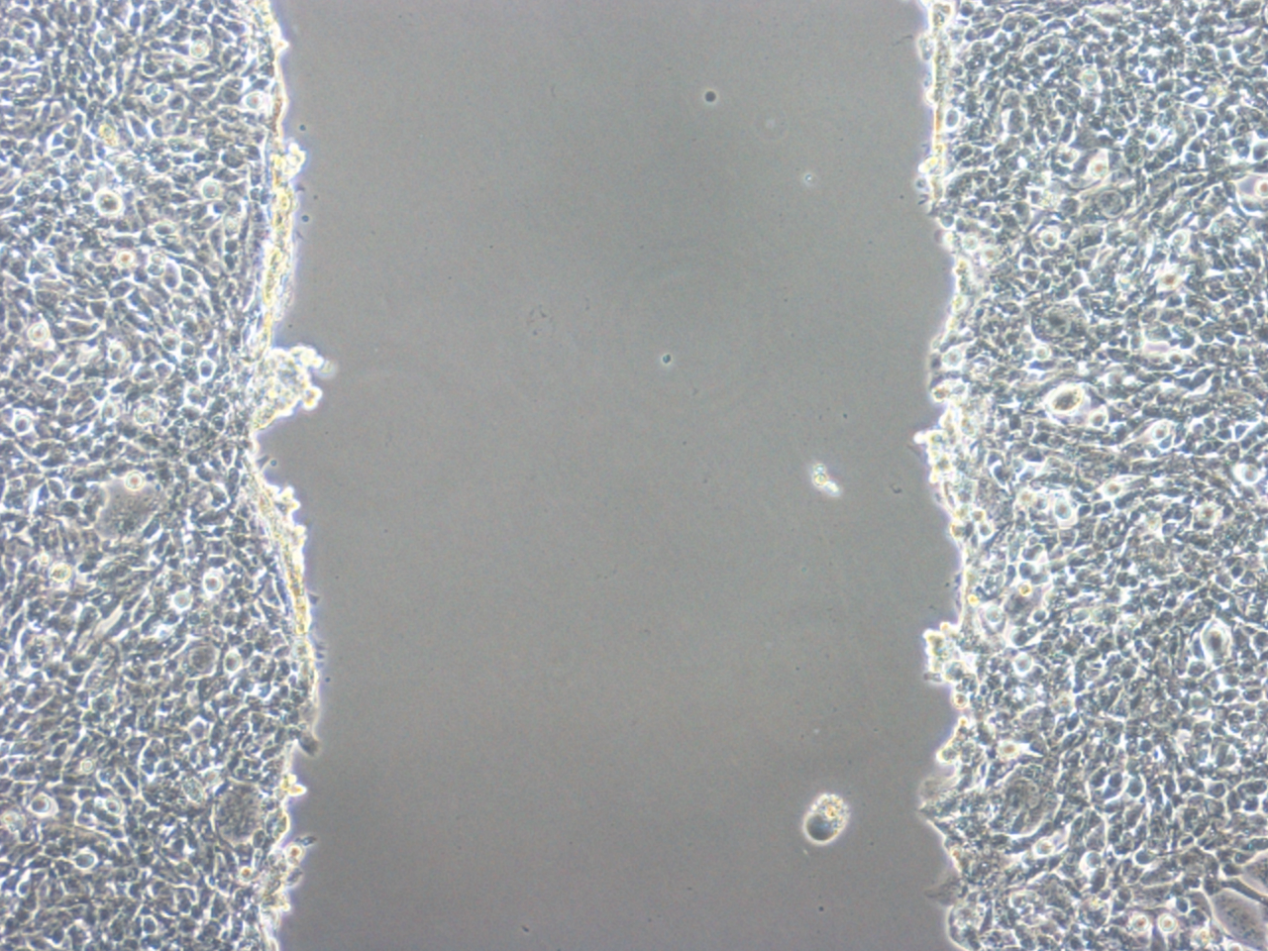


1. A172- si-COL3A1-24h


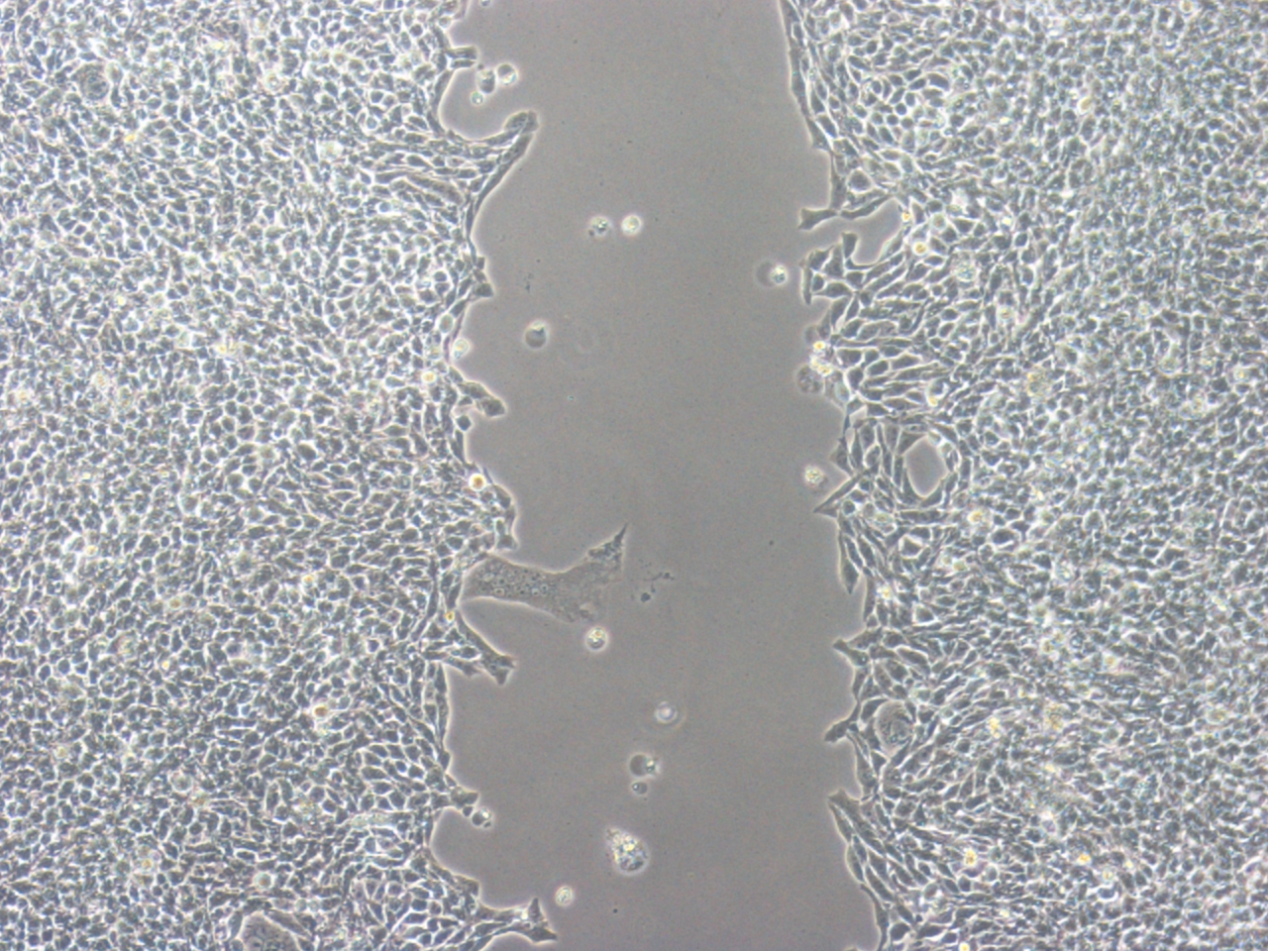


Transwell assay

1. SHG44-NC


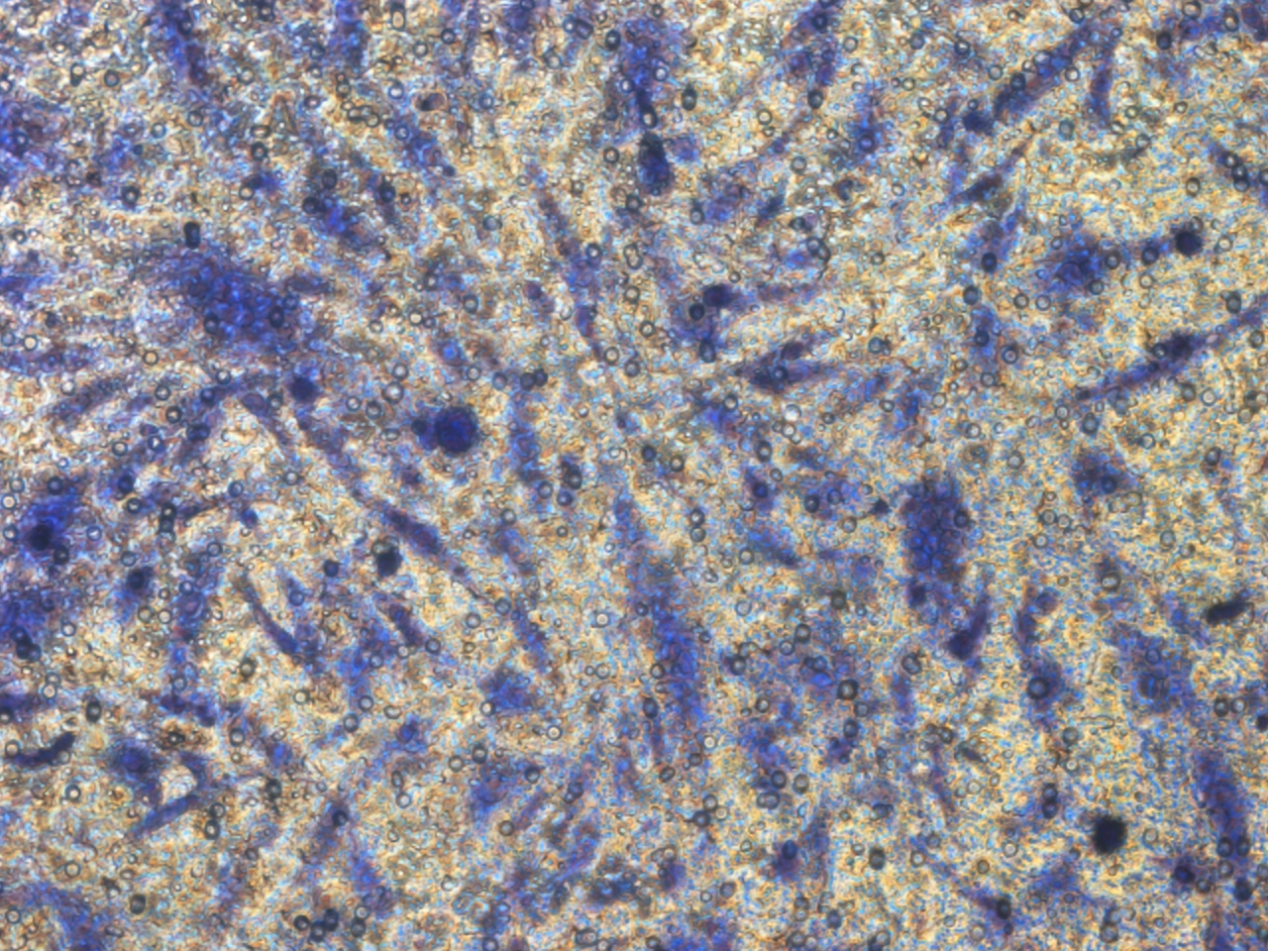


1. SHG44-si-COL3A1


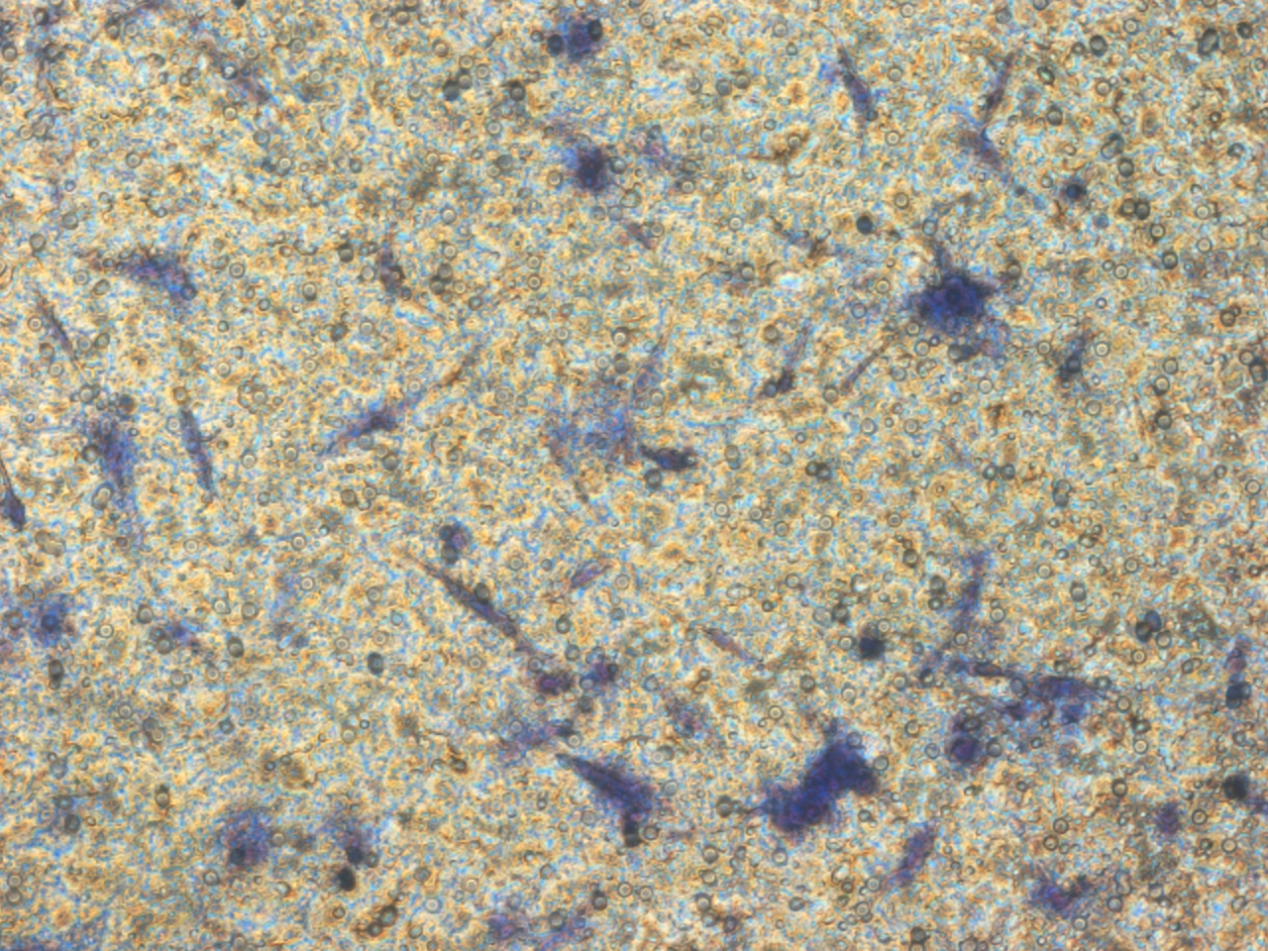


1. A172-NC


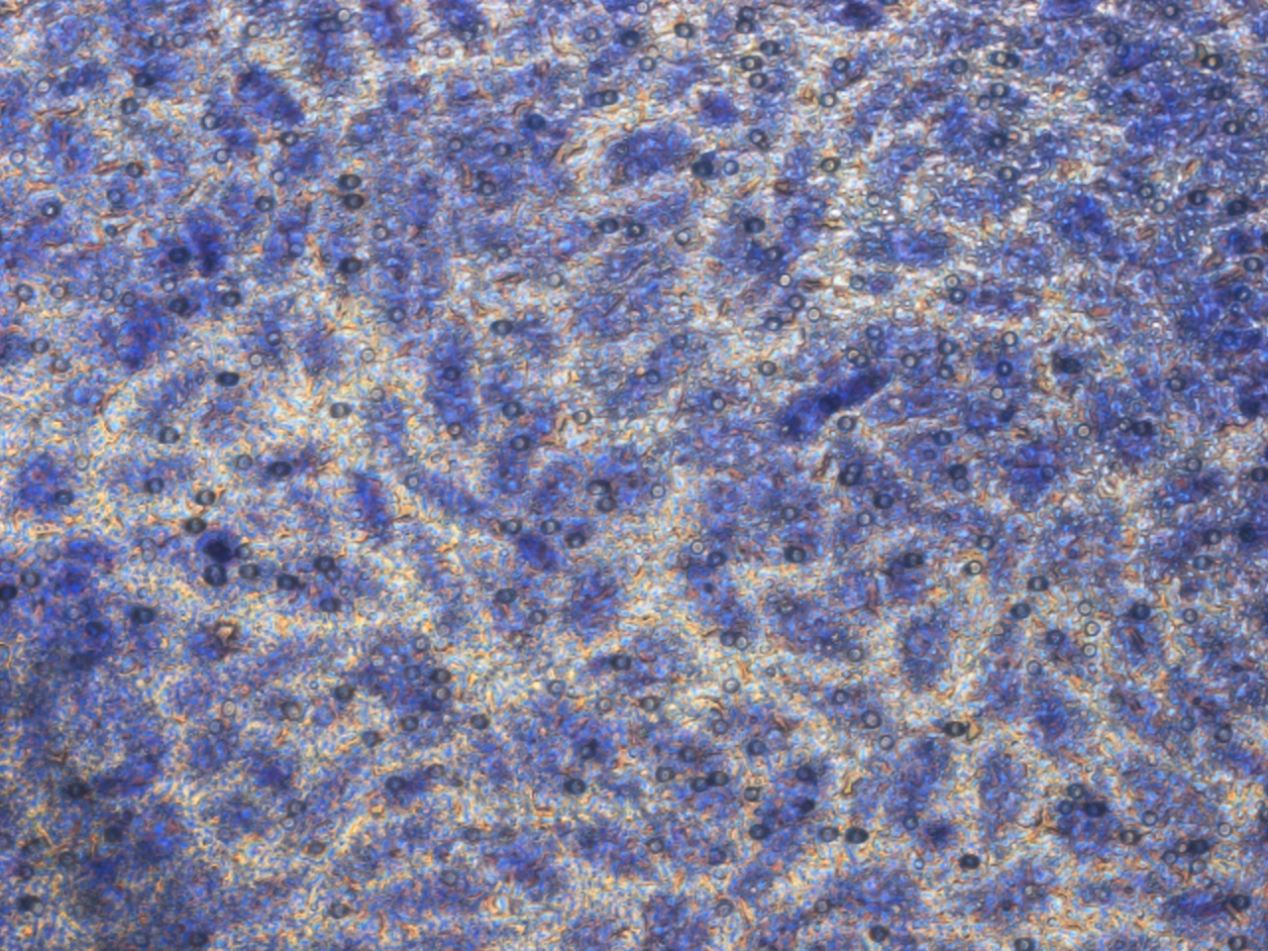


1. A172-si-COL3A1


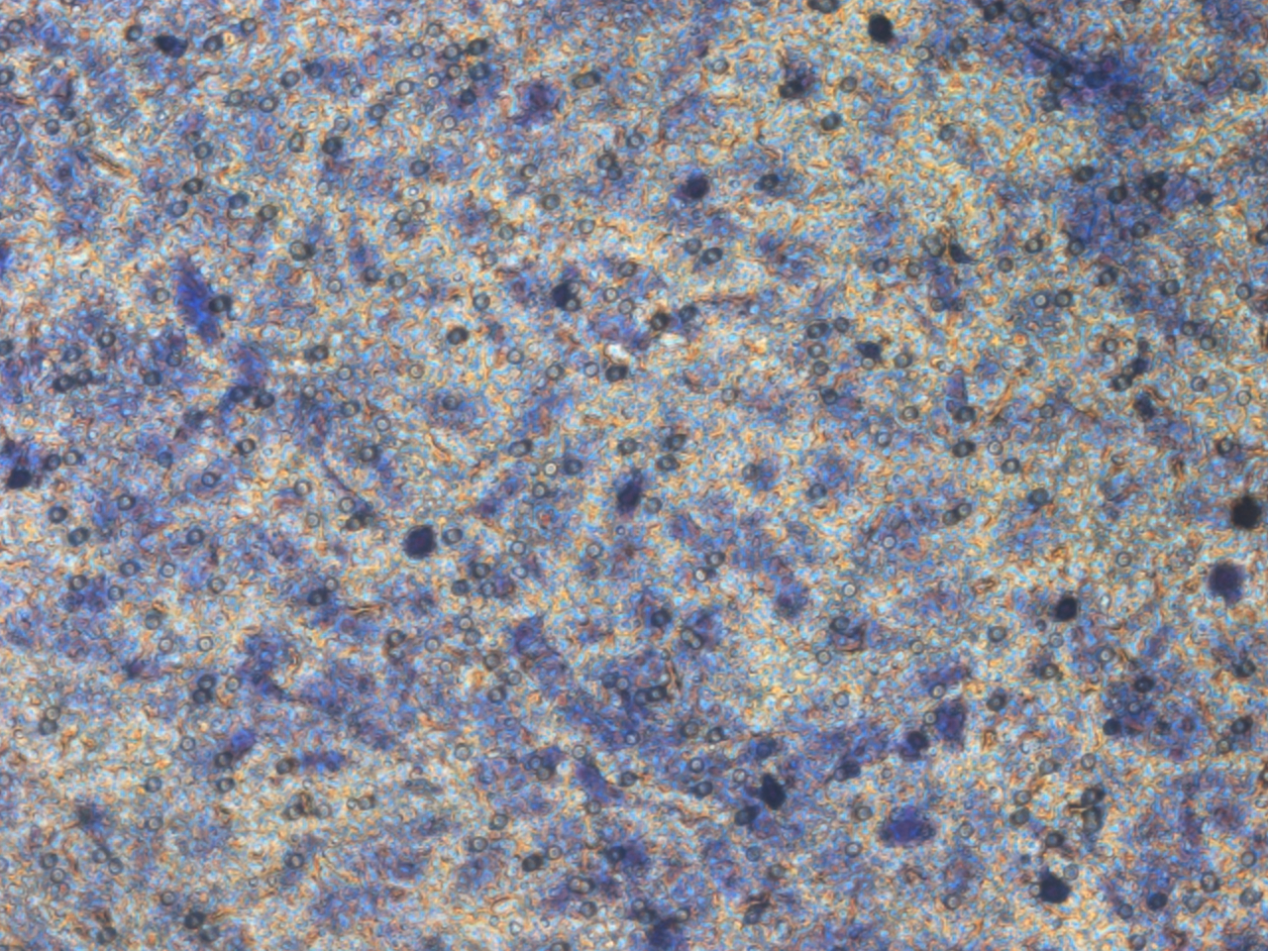


Western Blotting

1. GAPDH-SHG44-si-con-si-COL3A1


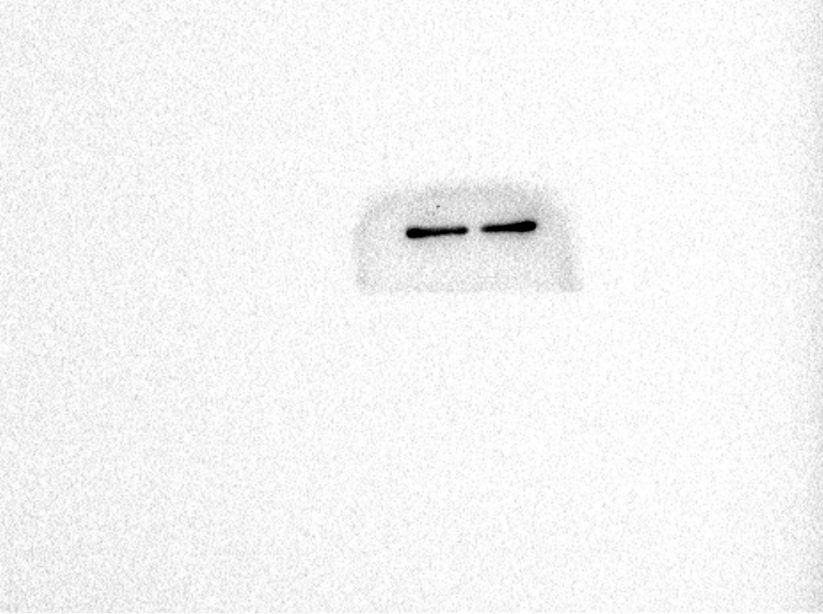


1. Vimentin-SHG44-si-con-si-COL3A1


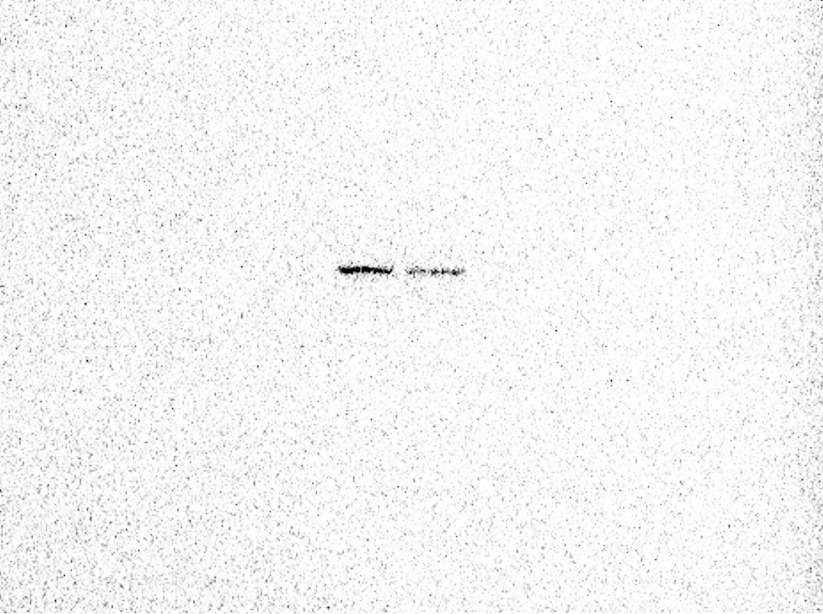


1. N-cadherin-SHG44-si-con-si-COL3A1


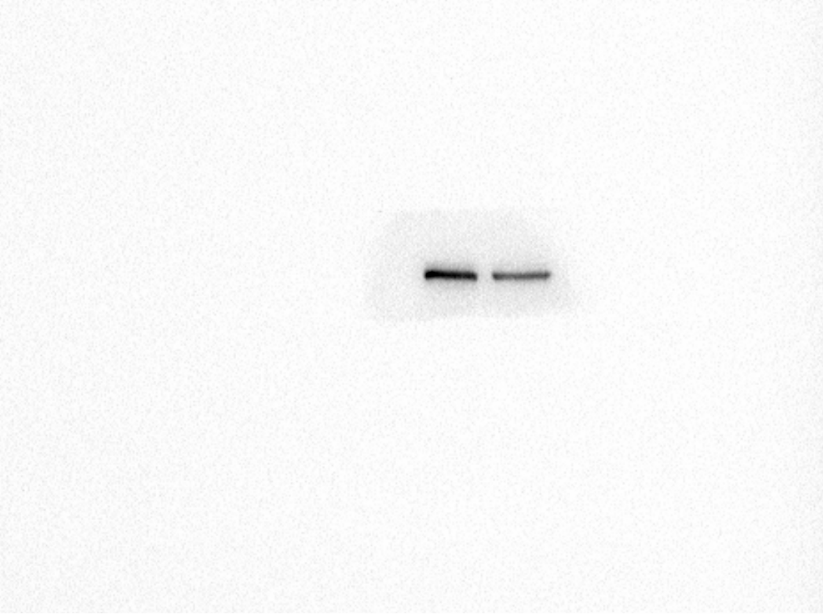


1. GAPDH-A172-si-con-si-COL3A1


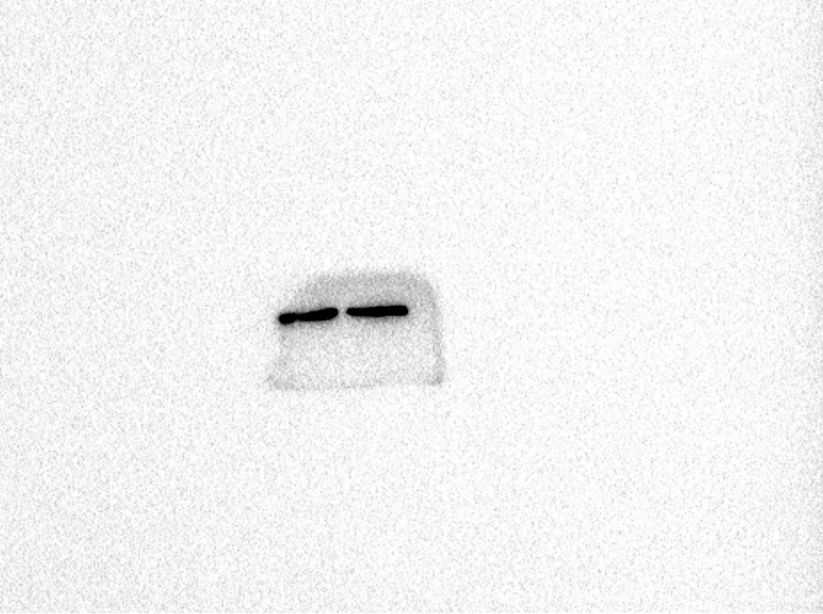


1. Vimentin- A172-si-con-si-COL3A1


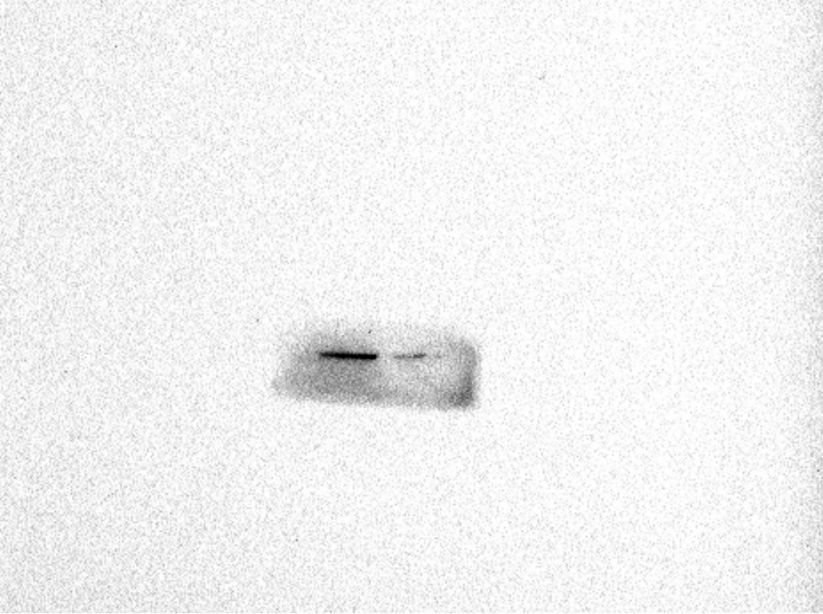


1. N-cadherin- A172-si-con-si-COL3A1


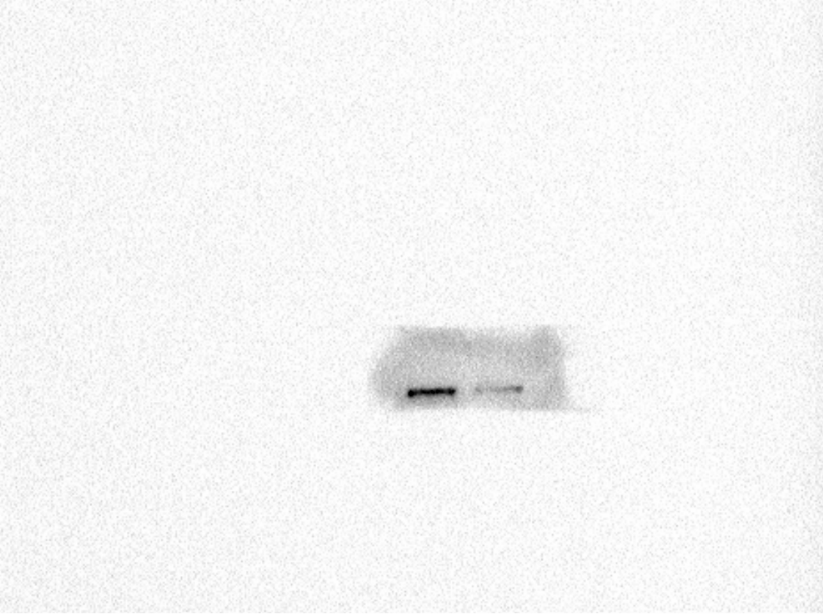

Supplement: Supplementary file 8 — Additional file 8: Fig. S8.. The original data of the experimental validation. [file 12935_2021_1982_MOESM8_ESM.docx]
